# Cold atmospheric plasma for preventing infection of viruses that use ACE2 for entry

**DOI:** 10.7150/thno.70098

**Published:** 2022-03-14

**Authors:** Peiyu Wang, Renwu Zhou, Rusen Zhou, Wenshao Li, Janith Weerasinghe, Shuxiong Chen, Bernd H. A. Rehm, Liqian Zhao, Francesca D. Frentiu, Zhifa Zhang, Kexin Yan, Mary Lor, Andreas Suhrbier, Derek J. Richard, Erik W. Thompson, Kostya Ken Ostrikov, Xiaofeng Dai

**Affiliations:** 1 Wuxi School of Medicine, Jiangnan University, Wuxi 214122, China.; 2 State Key Laboratory of Molecular Vaccinology and Molecular Diagnostics & Center for Molecular Imaging and Translational Medicine, School of Public Health, Xiamen University, Xiamen 361102, China.; 3 School of Biomedical Sciences, Queensland University of Technology, Brisbane 4059, Australia.; 4 Translational Research Institute, Woolloongabba, Queensland 4102, Australia.; 5 School of Chemistry and Physics, Queensland University of Technology, Brisbane 4000, Australia.; 6 State Key Laboratory of Electrical Insulation and Power Equipment, Center for Plasma Biomedicine, Xi'an Jiaotong University, Xi'an 710049, PR China.; 7 School of Biology and Environmental Science, Queensland University of Technology, Brisbane 4000, Queensland, Australia.; 8 Centre for Cell Factories and Biopolymers, Griffith Institute for Drug Discovery, Griffith University, Nathan, QLD 4111, Australia.; 9 The First School of Clinical Medicine, Southern Medical University, Guangzhou 510515, China.; 10 Centre for Immunology and Infection Control, Queensland University of Technology, Brisbane 4059, Australia.; 11 QIMR Berghofer Medical Research Institute, Herston QLD 4006, Australia.; 12 Australian Infectious Disease Research Centre, GVN Center of Excellence, Brisbane, Queensland, Australia.; 13 CAPsoul Biotechnology Company, Ltd, Beijing, China

**Keywords:** SARS-CoV-2, cold atmospheric plasma (CAP), Plasma activated medium (PAM), Angiotensin-converting enzyme 2

## Abstract

**Rational:** The mutating SARS-CoV-2 potentially impairs the efficacy of current vaccines or antibody-based treatments. Broad-spectrum and rapid anti-virus methods feasible for regular epidemic prevention against COVID-19 or alike are urgently called for.

**Methods:** Using SARS-CoV-2 virus and bioengineered pseudoviruses carrying ACE2-binding spike protein domains, we examined the efficacy of cold atmospheric plasma (CAP) on virus entry prevention.

**Results:** We found that CAP could effectively inhibit the entry of virus into cells. Direct CAP or CAP-activated medium (PAM) triggered rapid internalization and nuclear translocation of the virus receptor, ACE2, which began to return after 5 hours and was fully recovered by 12 hours. This was seen* in vitro* with both VERO-E6 cells and human mammary epithelial MCF10A cells, and *in vivo*. Hydroxyl radical (·OH) and species derived from its interactions with other species were found to be the most effective CAP components for triggering ACE2 nucleus translocation. The ERα/STAT3(Tyr705) and EGFR(Tyr1068/1086)/STAT3(Tyr705) axes were found to interact and collectively mediate the effects on ACE2 localization and expression.

**Conclusions:** Our data support the use of PAM in helping control SARS-CoV-2 if developed into products for nose/mouth spray; an approach extendable to other viruses utilizing ACE2 for host entry.

## Introduction

In the process of virus infection, the binding of viruses to host receptors leading to their entry into host cells is the first, critical and rate-limiting step^1^. The two main viral entry pathways, *i.e.*, receptor-mediated endocytosis^2^,^3^ and TMPRSS2-mediated membrane fusion^4^, ^5^, have facilitated the entry of all pandemic viruses from the past 20 years, SARS-CoV (2002)^2^,^5^, MERS-CoV (2013)^3^,^5^, and SARS-CoV-2 (2019)^4^ into host cells. Angiotensin-converting enzyme 2 (ACE2) plays a vital role in both pathways, especially for SARS-CoV^2^,^5^ and SARS-CoV-2^4^,^6^. The viral spike (S) protein binds to ACE2 to attach viruses on the host cell surface^4^, activating endocytosis or membrane fusion. Importantly, the UK variant-B.1.1.7 (Alpha), South Africa variant-B.1.351 (Beta), Brazilian variant-P.1 (Gamma) and Philippines variant-P.3 (Theta) lineage show increased transmissibility due to enhanced ACE2 binding affinity as a result of mutation N501Y^7^,^8^. Also, the mutation L452R carried by California coronavirus variant-B.1.427/B.1.429 (Epsilon) and Indian double mutant-B.1.617.1 (Kappa)^9^ exhibited enhanced interactions between ACE2 and the spike receptor binding domain (RBD)^10^. The high transmission rate of the India variant B1.617.2 (Delta) has been attributed to its high binding affinity to ACE2 as a result of three mutations in RBD, i.e., L452R, P681R and T478K^11^,^12^. The spike protein of the SARS-CoV-2 lineage C.37 (Lambda) has a unique pattern of 7 mutations (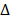
246-252, G75V, T76I, L452Q, F490S, D614G, T859N) where L452Q shares a similar impact on virus-cell interactions through ACE2, leading to substantially improved transmissibility^13^. Vaccines have been proven effective in controlling the morbidity and mortality of viral diseases by activating the generation of neutralizing antibody (NAb)^14^. However, SARS-CoV-2 can still be detected on nasal turbinates even after systemic NAb treatment^15^, suggesting that vaccines do not completely block virus infection. In fact, mutated viruses could partly (E484K, Alpha, Beta, Gamma, Epsilon, Eta, Iota, Zeta and Theta) and (E484Q, Kappa) or completely (S477N, Iota) escape from NAb-mediated neutralization^8^,^16^, and positive COVID-19 cases have been reported among vaccinated individuals^17^. The omicron variant, firstly being reported on Nov 25, 2021 and now imposing another round of global health threat, contains some deletions (e.g., 69-70 del, G142D/143-145 del) and over 30 mutations (e.g., T95I, K417N, T478K, N501Y, N655Y, N679K, P681H)^18^, which lead to enhanced transmissibility and extreme escape from immunological control^19^-^23^. Therefore, the establishments of further rapid, broad-spectrum anti-viral therapies, including ACE2-based preventive approaches, are urgently called for.

Cold atmospheric plasma (CAP), comprising partly ionized gas with a high electron temperature but low overall temperature, has been widely used in the biomedical sector^24^ for, e.g., skin disinfection^25^ and blood coagulation^26^, and has novel applications such as neural differentiation induction^27^ and emerging anti-cancer potential^28^-^36^. Its roles in modifying the cell surface for safe yet desirable cellular responses have been well documented^37^. It had been proven that direct CAP treatment could fragment the viral capsid protein and damage the RNA of norovirus (NoV)^38^, bacteriophage MS2^39^, immunodeficiency virus (HIV-1)^40^ and SARS-CoV-2 41, 42. CAP pre-treated host cell, monocyte-derived macrophages (MDM), could escape viral infection by down-regulating CD4 and CCR5^40^. In this study, we investigated whether direct CAP or indirect CAP in the form of plasma-activated medium (PAM, produced from the plasma-liquid interactions) is preventive against SARS-CoV-2 infection by decreasing ACE2 surface prevalence using SARS-CoV-2 and bioengineered pseudovirus containing ACE2-binding spike protein domains. The proposed strategy, i.e., through effective control of ACE2 cell membrane expression in tissues with high exposure risk such as nose, throat and lung^43^, may represent an emerging and promising approach against the outbreak of viral diseases relying on this receptor for host entry^44^.

By fabricating pseudoviruses with the ACE2-binding domain of spike protein, and conducting experiments using pseudoviral and SARS-CoV-2, we demonstrated the efficacy of PAM in protecting SARS-CoV-2 entry into Vero cells without cell toxicity. We found that CAP-triggered ACE2 relocation from cell surface to the nucleus via the ERα/STAT3(Tyr705) axis and reduced ACE2 expression through EGFR(Tyr1068/1086)/STAT3(Tyr705) signaling. Among the tested short- and long-lived reactive oxygen and nitrogen species (RONS) generated with CAP, hydroxyl radicals (·OH) and reactive species generated through its interactions with other species in PAM were found to be the most important CAP components triggering reduced ACE2 expression.

## Methods

### Cell culture

MCF10A cells are normal epithelial breast cells and used here as the* in vitro* cell model to investigate the effect of CAP on ACE2 in healthy epithelial cells. MCF10A immortalized diploid quasi-normal human mammary epithelial cells obtained from ATCC (Manassas, USA) were cultured in DMEM with 20 ng/mL EGF, 100 ng/mL Cholera Toxin, 5% horse serum, 10 µg/mL insulin, 1% streptomycin and 0.5 mg/mL hydrocortisone (Sigma, USA).

Vero E6 cells (African green monkey kidney cells) have abundant ACE2 expression and have been extensively used as cell-based virus infection models for SARS-CoV research since 2003^45^, and are thus used as the cell model for SARS-CoV-2 virus infection in this study. Vero E6 cells (ATCC C1008, ECACC, Wiltshire, England; Sigma Aldridge, St. Louis, MO, USA; CRL-1586, Manassas, USA) were cultured in RPMI-1640 with 10% fetal calf serum (FCS) (Sigma, USA). Cells were passaged with trypsin and stored under liquid nitrogen. Cells were routinely subjected to STR profile validation and confirmed negative for mycoplasma.

### Plasma-activated medium (PAM) generation

The CAP generation device was a typical high-frequency (1.7 MHz, 2-6 kV), atmospheric pressure Argon plasma jet (model kINPen 09) developed at the Leibniz-Institute for Plasma Science and Technology (INP Greifswald, Germany). CAP was streamed out of the quartz tube via the Argon gas pressure with a flow rate of 5.0 Standard L/min (power: < 3.5 W in the hand-held unit). Aliquots (1 mL) of medium without serum in 2 mL centrifuge tubes were activated by CAP for 5 s and 30 s, namely '5 s-activated' and '30 s-activated' PAM. PAM used for SARS-CoV-2 virus treatment was CAP-activated for 10 min and diluted to 10% before being transferred to the PC3 lab. The distance between the kINPen jet and the liquid surface was 1 cm.

### Staining reagents and antibodies

Primary antibodies: SARS-CoV-2 Spike S1 Antibody (HC2001, GenScript Biotech, USA), ACE2 for Western blot (WB; sc-20998, Santa Cruz, USA), ACE2 for immunofluorescence (IF) (sc-73668, Santa Cruz, USA), STAT3 (12640, Cell Signalling, USA), p-STAT3 (Try705) (9145, Cell Signalling, USA), EGFR (sc-120, Santa Cruz, USA), p-EGFR(Tyr1068) (44788G, Thermo-Fisher Scientific, USA), p-EGFR(Tyr1086) (369700, Thermo-Fisher Scientific, USA), ERα (ab14020, Abcam, USA), β-actin (sc-8432, Santa Cruz, USA). Secondary antibodies: goat anti-human IgG Alexa Fluor 555 (A-21433, ThermoFisher, USA), goat anti mouse IgG-FITC (sc-2010, Santa Cruz, USA), goat anti-chicken IgY H&L Alexa Fluro 647 (ab150175, Abcam, USA), goat anti-mouse IgG H&L Alexa Fluro 647 (ab150115, Abcam, USA), goat anti-mouse IgG-HPR (sc-2031, Santa Cruz, USA), goat anti-rabbit IgG H&L-HRP (ab6721, Abcam, USA). Phalloidin labelled with Alexa Fluor 647 for F-actin staining (A22287, ThermoFisher, USA). Hoescht 33342 for nuclear (DNA) staining (H3570, ThermoFisher, USA).

### Western blot

Proteins were collected in RIPA buffer with phosphatase inhibitor (Thermo-Fisher Scientific), and levels normalized after Pierce BCA Protein Assay Kit (Thermo-Fisher Scientific) analysis. Each protein sample (10 mg) was diluted with 4×loading buffer, separated by a 10% SDS-PAGE gel, and wetly transferred to polyvinylidene fluoride (PVDF) membranes (Thermo-Fisher Scientific). Membranes were blocked (3% BSA in (Tris-HCl buffer and Tween; TBST)) for 1 h. The primary antibodies were diluted (3% BSA in TBST) at a ratio of 1:1000. All membranes were incubated with primary antibodies overnight at 4 °C with shaking. Membranes were washed for 10 min using TBST buffer three times with shaking, followed by incubation with HRP-conjugated secondary antibodies for 2 h at 4 °C with shaking. The secondary antibodies were diluted (3% BSA in TBST) at a ratio of 1:4,000. Membranes were washed 3×10 min with TBST and incubated by enhanced chemiluminescence (ECL) (Bio-Rad) for 5 min. The chemical fluorescence from blots was photographed and scanned by ChemiDoc XRS+ system (Bio-Rad). The images were analyzed and normalized by Image Lab program (Bio-Rad).

### SARS-CoV-2 stock production and titration

SARS-CoV-2 infection studies at QIMR Berghofer were conducted in a dedicated PC3 (BSL3) suite, with safety approval from the QIMR Safety Committee (P3600). The SARS-CoV-2 virus was isolated from a patient and was a kind gift from Queensland Health Forensic & Scientific Services, Queensland Department of Health; the isolate (hCoV-19/Australia/QLD02/2020) has been sequenced, and is available at GISAID (https://www.gisaid.org/). Virus stock was generated by infecting Vero E6 and after 3 days culture supernatant was clarified by centrifugation at 3000 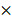 g for 15 min at 4°C, and was aliquoted and stored at -80°C. Virus titer (10E6.79 TCID_50_/mL virus) was determined using standard CCID50 assay by infecting Vero E6 cells with 10-fold serial dilutions of virus stock and measuring cytopathic effect with titer calculation by the method of Spearman and Karber. Virus was determined to be mycoplasma free^46^ and FCS used for culture determined to be endotoxin free^47^.

### SARS-CoV-2 virus experiment

Vero E6 cells were seeded in a 96-well-plate at 10,000/well (100 μL) for 24 h prior to PAM treatment. 10% FCS RPMI-1640 media was removed and replaced with 10% PAM (pre-warmed to 37°C for 5 min) for 10 min. PAM was removed and changed to the 10% FCS RPMI-1640 medium. The virus stock was diluted to 1/10^2^, 1/10^3^, 1/10^4^, 1/10^5^ or 1/10^6^ in 10% FCS RPMI-1640 medium and added to cells (quadruplicate wells for each treatment group) for 2 additional hours, after which the medium was removed, cells were washed twice using phosphate buffered saline (PBS), and the cultures were incubated in 10% FCS RPMI-1640 medium, which also served as a no-virus control. Wells were fixed by 4% glutaraldehyde 24 h after virus addition, stained for ACE2, SARS-CoV-2 virus, actin, DNA as described under immunofluorescence and subjected to scanning electron microscopy (SEM). This SARS-CoV-2 virus experiment was independently conducted three times.

### Immunofluorescence

For SARS-CoV-2 virus analysis, cells in four representative wells were fixed and permeabilized in 0.2% Triton X-100 for 5 min followed by blocking (3% BSA in PBS) for 1 h. Cells were then incubated with both ACE2 and SARS-CoV-2 Spike S1 antibodies diluted 1:1000 in PBS containing 3% BSA overnight, and washed 3×10 min with PBS. Goat anti-human IgG Alexa Fluor 555 (A-21433) and goat anti mouse IgG-FITC (sc-2010) were used as secondary antibodies at a dilution ratio of 1:4000 using PBS containing 3% BSA. After 2 h incubation, cells were washed 3×10 min with PBS and incubated with Phalloidin at the room temperature for 30 min, and again washed 3×10 min with PBS and incubated with Hoescht 33342 dye for 5 min. High-throughput analysis was performed using InCell 6500HS (60×, 120 fields per well) and quantified by IN Carta analysis software.

For analysis of CAP and PAW effects on ACE2 levels and location, MCF10A or Vero E6 cells were plated at 10,000 cells per well for 12 h. After treatment, cells were fixed with 4% paraformaldehyde in PBS for 10 min at 4 °C. Cells were first permeabilized in 0.2% Triton X-100 for 5 min and then blocked (3% BSA in PBS) for 1 h, followed by incubation with primary antibodies overnight. After incubation, cells were washed 3×10 min with PBS before the addition of secondary antibodies followed by incubation at the room temperature for 1 h. Nuclear DNA was stained with Hoechst 33342 for 5 min at 1 μg/mL. Delta Vision Elite Live Imaging Microscope (Applied Precision, GE Healthcare Lifesciences, Parramatta NSW, AUS) and softWoRx analysis software (Applied Precision, USA) were used for cell imaging.

### Scanning electron microscopy

All samples were fixed in 4% glutaraldehyde (Sigma-Aldrich, USA) in PBS buffer. Subsequently, an increasing gradient of ethanol solutions (0%, 30%, 40%, 50%, 60%, 70%, 80%, 90% and 100%) was implemented for dehydration followed by 100% Hexamethyldisilazane (HMDS) (Merck, Australia) for 30 mins. All samples were dried in a biosafety hood for 24 h. Finally, all samples were loaded onto 12.6 mm SEM mounts (ProSciTech, AU) using 12 mm double-sided carbon tabs (ProSciTech, AU) and sputtered with a 10-nm-thick gold layer using Cool Sputter Coater Leica EM SCD005 (Leica, DE). SEM analysis was performed under 5 kV voltage and 8 A current using a JEOL JSM-7001F (JEOL Ltd. JP) at magnifications of ×7,500, ×10,000 and ×15,000.

### ROS scavenging assay

Reactive species trapping tests were carried out to investigate the respective roles of different active species generated in PAM. Sodium pyruvate (10 mM), uric acid (100 μM), mannitol (200 mM), tiron (20 mM), hemoglobin (20 mM), monopotassium phosphate(1 mM) were purchased from Signa-Aldrich (Australia) and used to trap H_2_O_2_, O_3_, ·OH, ·O_2_^-^, ·NO and e^-^, respectively^48^-^50^. MCF10A cells (10,000 cells per well) were seeded in the 96-well plate and cultured for 24 h, with 5,000 cells per well. 1 mL DMEM medium was activated by CAP for 30 s to prepare PAM following the protocol described in the 'PAM generation' section. PAM was divided into 7 tubes, 90 µL per tube. 10 µL of each ROS scavenger was added to 90 µL PAM followed by thorough mixing and 1 min incubation. 50 µL of each mixture was then added to MCF10A cells for 30 s followed by immunofluorescence imaging of ACE2. All scavengers were proven non-toxic at the working concentrations used in the assays^51^.

Sodium pyruvate is not a specific scavenger for H_2_O_2_ but can also react with peroxynitrite (ONOO^-^)^52^. Thus, inhibition of any reaction by pyruvate can be explained by either the action of H_2_O_2_ or ONOO^-^.

### ROS quantification assay

The titanium sulfate method was used to measure H_2_O_2_, where H_2_O_2_ and titanium oxysulfate (TiOSO_4_) could react and generate a yellow-colored complex (pertitanic acid) that can be quantified by spectroscopy at 410 nm^53^. ·OH concentrations were determined using benzene‐1,4‐dioic acid (TA) and measured by the fluorescence assay, where ·OH radicals react with TA to form 2-hydroxyterephthalic acid (HTA) with an irradiation wavelength of 310 nm and a fluorescence emission wavelength of 425 nm^54^. A multiparameter photometer (Hanna Instruments, HI83399) was used to determine the O_3_ concentration using a certified ozone reagent kit from the supplier. The Griess Reagent method was used to quantify NO^2-^; and a nitrate specific ion electrode was used to quantify NO^3-^, with an Ionic Strength Adjuster (NH_4_)_2_SO_4_ (2 mol/L) being added to maintain a constant ionic strength before detection^55^. A spin trap comprised of a complex of N-methyl-D-glucamine dithiocarbamate (MGD) and iron (II) ion was used to react with ·NO to form (MGD)_2_Fe^2+^-NO adducts, which were detected by ESR^51^.

### Plasmids for biological assembly of SARS-CoV-2 protein coated polyester particles

The following DNA fragments encoding the SARS-CoV-2 proteins N (nucleocapsid) and RBD (receptor binding domain derived from spike protein) had been previously synthesized by Biomatik (Canada) and codon optimized for *E. coli* strains:

#### N protein

SDNGPQNQRNAPRITFGGPSDSTGSNQNGERSGARSKQRRPQGLPNNTASWFTALTQHGKEDLKFPRGQGVPINTNSSPDDQIGYYRRATRRIRGGDGKMKDLSPRWYFYYLGTGPEAGLPYGANKDGIIWVATEGALNTPKDHIGTRNPANNAAIVLQLPQGTTLPKGFYAEGSRGGSQASSRSSSRSRNSSRNSTPGSSRGTSPARMAGNGGDAALALLLLDRLNQLESKMSGKGQQQQGQTVTKKSAAEASKKPRQKRTATKAYNVTQAFGRRGPEQTQGNFGDQELIRQGTDYKHWPQIAQFAPSASAFFGMSRIGMEVTPSGTWLTYTGAIKLDDKDPNFKDQVILLNKHIDAYKTFPPTEPKKDKKKKADETQALPQRQKKQQTVTLLPAADLDDFSKQLQQSMSSADSTQA.

#### RBD protein

RVQPTESIVRFPNITNLCPFGEVFNATRFASVYAWNRKRISNCVADYSVLYNSASFSTFKCYGVSPTKLNDLCFTNVYADSFVIRGDEVRQIAPGQTGKIADYNYKLPDDFTGCVIAWNSNNLDSKVGGNYNYLYRLFRKSNLKPFERDISTEIYQAGSTPCNGVEGFNCYFPLQSYGFQPTNGVGYQPYRVVVLSFELLHAPATVCGPKK.

All clonings had been carried out using the *E. coli* Top10 strain (Table 1) following the protocol described in Reference 56. The target DNA sequences of all pET plasmids were confirmed by the Griffith University DNA Sequencing Facility (Griffith University, Australia). Three genes, phaA, phaB, and phaC encoding enzymes PhaA, PhaB, and PhaC were required for polyester particle formation. The plasmid pMCS69 contains the genes phaA and phaB, and the pET plasmid contains the gene phaC. Both pMCS69 and pET-14b phaC- SARS-CoV-2 genes were transformed into the endotoxin-free production strain, *ClearColi* BL21(DE3) (Table 1).

### Bacteria growth condition enabling production of protein-coated polyester particles

The cloning strain *E. coli* Top 10 was grown in Luria broth (LB broth) (10 g/L Peptone, 5 g/L Yeast Extract, 5 g/L Sodium Chloride) supplemented with ampicillin (100 µg/mL) at 37 ºC. The production strain ClearColi BL21(DE3) was incubated in LB Miller media (10 g/L Peptone, 5 g/L Yeast Extract, 10 g/L Sodium Chloride) supplemented with 1% (w/v) glucose, ampicillin (100 µg/mL), and chloramphenicol (50 µg/mL). 2% overnight cell cultures were inoculated into LB broth and incubated at 37 ºC, 200 rpm for approximately 3 h to allow the culture to reach an optical density OD600 between 0.5 ~ 0.8, where cells were at the log growth phase providing optimal bacterial physiology for high level protein production. Protein production was induced using 1 mM isopropyl b-D-1-thiogalactopyranoside and cells grew continuously at 25 ºC for 48 h to enable efficient formation of polyester particles that were densely coated with SARS-CoV-2 derived proteins.

### Particle extraction

The procedures of polyester particle isolation and purification are described elsewhere^58^,^60^. Briefly, cells were harvested by centrifugation (8,000 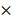 g for 15 min), resuspended in 0.5 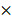 lysis buffer (10 mM Tris, 5 mM EDTA, 0.04% w/v SDS, pH 7.5), and subjected to mechanical disruption using a microfluidizer M-110P (Microfluidics, USA). Particles were washed 3 times with 0.5 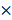 lysis buffer after cell disruption and then stored in Tris buffer (10 mM Tris, pH 7.5) with 20% ethanol at 4 ºC.

### Particle characterization

Protein profiles of purified polyester particles displaying SARS-CoV-2 antigen was analyzed using densitometry. Particle samples were loaded on 10% Bis-Tris gel and target protein purity in the particle fraction was analyzed using Image Lab Software (Bio-Rad Laboratories, USA). The recombinant target protein band was excised from the gel and subjected to protein identification using Quadrupole Time of Flight mass spectrometry (Q-TOF/MS), performed at the Mass Spectrometry Facility, UQ Centre for Clinical Research (Queensland, Australia). Morphology and size of purified SARS-CoV-2 particles and *ClearColi* BL21(DE3) cells harboring the particles were analyzed by transmission electron microscopy (TEM) as previously described^58^,^61^,^62^ in the Manawatu Microscopy & Imaging Centre (Massy University, New Zealand). The ζ-potential and size distribution of SARS-CoV-2 antigen displaying polyester particles in 10mM Tris buffer pH7.5 were measured by the Zetasizer Nano ZS (Malvern Panalytical, UK).

### *In vitro* SARS-CoV-2 polyester particles and ACE2 binding

High-binding plates (Greiner Bio-One, Germany) were coated overnight at 4 °C with 100 μL of 2 μg/mL purified polyester particles diluted in PBS containing 0.05% (v/v) Tween 20, pH7.5 (PBST). The plain PhaC particle and purified soluble glycosylated S1 protein containing the RBD domain produced using baculovirus expression systems^63^ provided the negative and positive controls, respectively. The plate was washed six times with PBST and then blocked with 3% BSA in TBST at 25 ºC for 1 h. After six washes, ACE2 (human) Fc fusion (HEK293) (Aviscera Bioscience Inc, USA) diluted with PBST was added to the plate and incubated for 1 h at 25 ºC. To detect and quantify bound ACE2-Fc, the plate was incubated with protein A-HRP (Abcam, UK) for 1 h at 25 ºC after six washes with PBST. O-phenylenediamine substrate (Abbott Diagnostics, IL, USA) was added onto the plate for signal development. The result was measured at 490 nm with an ELx808iu ultramicrotiter plate reader (Bio-Tek Instruments Inc., USA). The ACE2 binding assay was carried out at the Centre for Cell Factories and Biopolymers, Griffith Institute for Drug Discovery, Griffith University, Queensland, Australia.

### Effects of CAP on nanoparticle binding to cells

VERO-E6 cells were plated in 96-well-plates at 10,000 cells per well for 24 h, and supplied with 0.5, 1, 2, 3, 5, 7, 10, 20 or 50 µL of Nile Red-labelled particles A, B or C, for 5 min. The medium was removed, and cells were stained by Hoechst 33342. Cells were scanned by Incell 6500HS and the intensity of Nile Red was calculated by IN Carta Analysis.

For CAP treatment, cells seeded as above were treated with or without 5 s, 30 s or 1 min CAP, or with 5%, 10%, 30%, 50% or 100% PAM incubation for 1 min. The medium was refreshed with 50 µL DMEM containing 10 µL particles and incubated for 5 min. Cells were stained and analyzed for NP uptake as above.

### Live/dead assay

Cells were plated in a 96-well plate for 24 h at 10,000 cells/well. Propidium iodide (10 μg/mL) and Hoechst 33342 (1 μg/mL) were added 30 min prior to imaging following cell CAP exposure or PAM treatment. Cells were imaged using an InCell 6500HS (10× objective), and live/dead cell analysis was performed using IN Carta analysis software.

### Annexin V/PI assay

Trypsinized cells were blocked with serum, transferred to a 1.5 mL centrifuge tube, and washed 3 times as per the Promega Annexin V-FITC Apoptosis Detection Kit (United Bioresearch; Dural NSW, Australia) guidelines. Before staining, cells in each group were re-suspended to 4×10^5^ cells in 400 μL pending buffer, and incubated with 488-Annexin antibody (1:40) for 15 min in the darkness. Cells were then washed and re-suspended for 3 times, and stained using propidium iodide diluted at a ratio of 1:20 using pending buffer. Cell fluorescence was measured using the Gallios flow cytometer system and analyzed using Kaluza software (Beckman, Lane Cove NSW, Australia).

### Cell cycle assay

MCF10A and Vero E6 cells were seeded in 6-well-plates at a concentration of 2
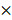
10^5^ cells per well for 24 hours. After PAM treatment, cells were detached from plates by trypsin and the cell suspension was centrifuged at 500 rpm for 5 min. After 15 min fixation in 70% cold ethanol, cells were washed twice by PBS and collected by centrifuge. Cells were adjusted to 2
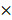
10^5^ cells/mL and stained by Hoechst 33342 (200 μL, 5 µg/mL). After staining, cells' suspension from different samples were analyzed using a flow cytometer Cytoflex (Beckman Coulter Life Sciences, USA) at a flow rate of 60 μL/min and excitation of 450 nm. Data were collected in 20,000 particle units and used to form the univariate flow karyotype histogram of fluorescence density signals, and analyzed by the FlowJo software to show the percentage of nuclei in the G1, S, and G2/M phases.

### Animal experiment

All animal experiments were performed in accordance with the National Institute of Health Guide for the Care and Use of Laboratory Animals and were approved by the Animal Laboratory Center of Jiangnan University.

SUM159PT cells suspended in PBS were injected subcutaneously in the right forelimbs of 10 female BALB/c mice aged 4 weeks with the weights of 16 ± 2 g on the first day, and each mouse was injected with 1×10^6^ cells. Mice were evenly divided into 2 groups, i.e., SUM159PT_control group (receiving untreated medium), SUM159PT_CAP group (receiving 10% PAM). The first treatment was performed when the diameter of a tumor reached 5 ± 0.5 mm, which was calculated following Equation 1


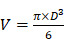
 , (1)

where '
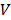
', '
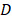
' each represents the volume and the diameter of the tumor, respectively. Mice were anesthetized with ketamine (10 mg/ml) intraperitoneally before each treatment. The injection volume was 10 μL/g of the mouse body weight. 10% PAM was subcutaneously injected at two sites of the tumor for each mouse with 100 μL/site. This treatment was repeated every 6 days. Tumors were dissected after the sacrifice of the mice 39 days from the day that mice were inoculated with tumor cells.

### Immunohistochemistry staining

The 5 μm thick paraffin sections were deparaffinized in xylene and rehydrated in ethanol at different gradients (100%, 100%, 95%, 70% in sequence). Tissue slices were incubated in 3% H_2_O_2_ for 20 min to inactivate endogenous peroxidase. After being heated in 10 mM citrate buffer for 15 min, tissue sections were incubated with a recombinant primary antibodies against glycolyzed ACE2 (1:200, ab108252, Abcam) overnight at 4°C. Corresponding secondary antibody was added and incubated for 1 h at the room temperature. Images were observed with Pannoramic MIDI (3DHISTECH Ltd, Budapest, Hungary).

### Statistical analyses

Statistical tests were performed by Graph Pad Prism V8 or SPSS Statistics 23. Data was deemed parametric if differences in variance was <4, skewness was >-2 and kurtosis was <2, and was analyzed using Student's two-tailed t-tests assuming unequal variance, otherwise the non-parametric Kolmogorov-Smirnov or Kruskal Wallace tests were used. P values > 0.05 were considered insignificant, i.e., “*” representing p < 0.05, “**” representing p < 0.01, “***” representing p < 0.001.

### Bioinformatic analyses

Differentially expressed genes were identified using R packages 'limma' and 'DESeq2', where genes with |fold change| > 1 and adjusted p value < 0.05 were selected. KEGG pathway enrichment analysis was conducted for all differentially expressed genes, as well as upward and downward regulated genes using the R package 'clusterProfiler'. The top 25 enriched pathways that satisfied adjusted p < 0.05 were selected for plotting.

## 3. Results

### Plasma-liquid interactions: ROS/RNS chemistry

**Figure 1:**
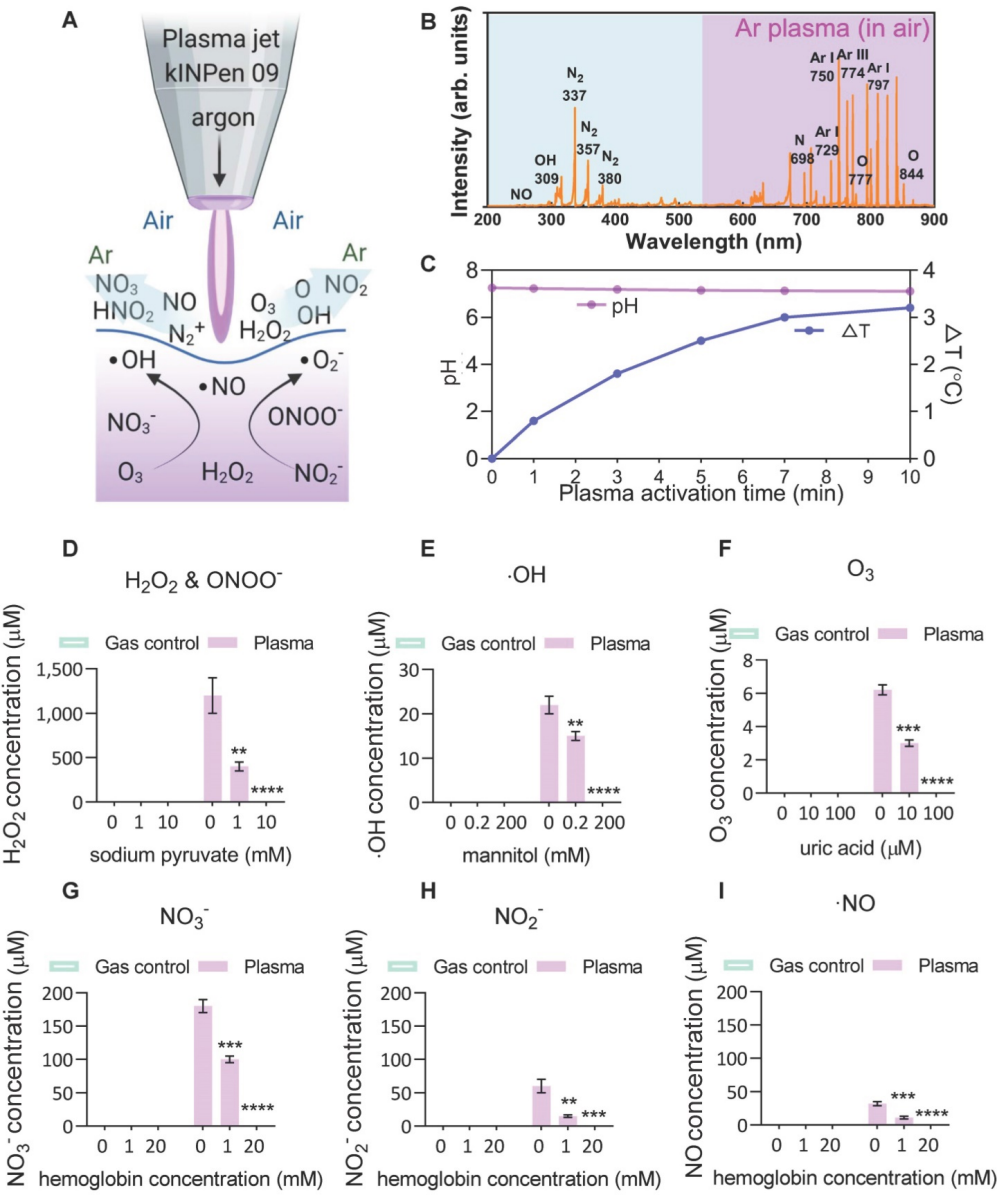
*Scheme of the plasma-liquid interactions and ROS/RNS fingerprints.* (*A*) Representation of the atmospheric pressure plasma jet kINPen used in this study, and schematic of the ROS/RNS produced at the gas-liquid interface; (*B*) Optical emission spectroscopy (OES) of the plasma jet showing different types of reactive atoms and molecules such as reactive nitrogen species (RNS), hydroxyl radicals (·OH), and atomic oxygen (O); (*C*-*I*) major reactive species analyzed in the control and CAP-treated PBS in the presence or absence of different scavengers including hydrogen peroxide, nitrite, nitrate, nitric oxide, ozone and hydroxyl radical. Mannitol, uric acid, hemoglobin and sodium pyruvate were used to quench hydroxyl radical, ozone, nitric oxide, and H_2_O_2,_ respectively. Data are representative of at least three experiments; statistical analysis was performed using one-way ANOVA (* *p* < 0.05; ** *p* < 0.01; *** *p* < 0.001).

To investigate the plasma-derived RONS as a therapy against ACE2-mediated virus entry, an atmospheric pressure argon plasma jet operated in open-air was employed to mimic a multi-ROS/RNS environment (Figure 1*A*). Optical emission spectroscopy (OES) was used to characterize the fingerprints of ROS/RNS in gaseous plasmas and PAM, where the discharge was found to be enriched with the second positive system of nitrogen, argon bands, hydroxyl radical and atomic oxygen (O) (Figure 1*B*). The produced argon plasma jet was subsequently used to treat a liquid, where the treatment of gas-only (plasma-off) served as the control. No reduction in solution pH but modest changes (~3 °C) in solution temperature was observed with a 10-min CAP exposure (Figure 1*C*). Subsequently, the analysis of commonly discussed primary and secondary reactive species in the liquid was performed in the absence or presence of specific scavengers. For hydrogen peroxide (H_2_O_2_), the argon plasma-liquid interactions yield this secondary oxidant mostly derived from hydroxyl radicals with a concentration of over 1 mM, and the presence of sodium pyruvate scavenger reduced the significant amounts of H_2_O_2_ or ONOO^-[52]^ (Figure 1*D*). The generation of ∙OH radicals, the most reactive species with an oxidative potential of -2.8 V, was also observed and confirmed by adding the mannitol scavenger (Figure 1*E*). Although they have a very short lifetime, ·OH radicals can be continuously produced from the secondary reactions (H_2_O_2_ →∙OH + ·OH, O_3_ + H_2_O → 2·OH + O_2_, O_3_ + HO_2_ → ·OH + 2O_2_)^64^. Compared with other species, ozone, which can be effectively scavenged by the uric acid scavenger, was less prominent after plasma activation, probably due to its low water solubility (Figure 1*F*). For N-containing species (nitrate, nitrite and nitric oxide), the oxidative environment favoured the generation of the most stable NO_3_^-^ (> 150 μM) and relatively stable but bio-reactive NO_2_^-^ (> 50 μM) (Figure 1*G***-***I*). A similar trend was observed with the addition of hemoglobin (Figure 1*G***-***I*), indicating that the secondary aqueous reactions of NO are important sources of the nitrate and nitrite produced in PAM. These results suggest that the CAP-liquid interactions can create a multiple oxidative solution environment rich in highly-reactive RONS, inherently linked to the biological consequences of CAP-based therapy.

### CAP effectively prevents cells from SARS-CoV-2 infection

**Figure 2:**
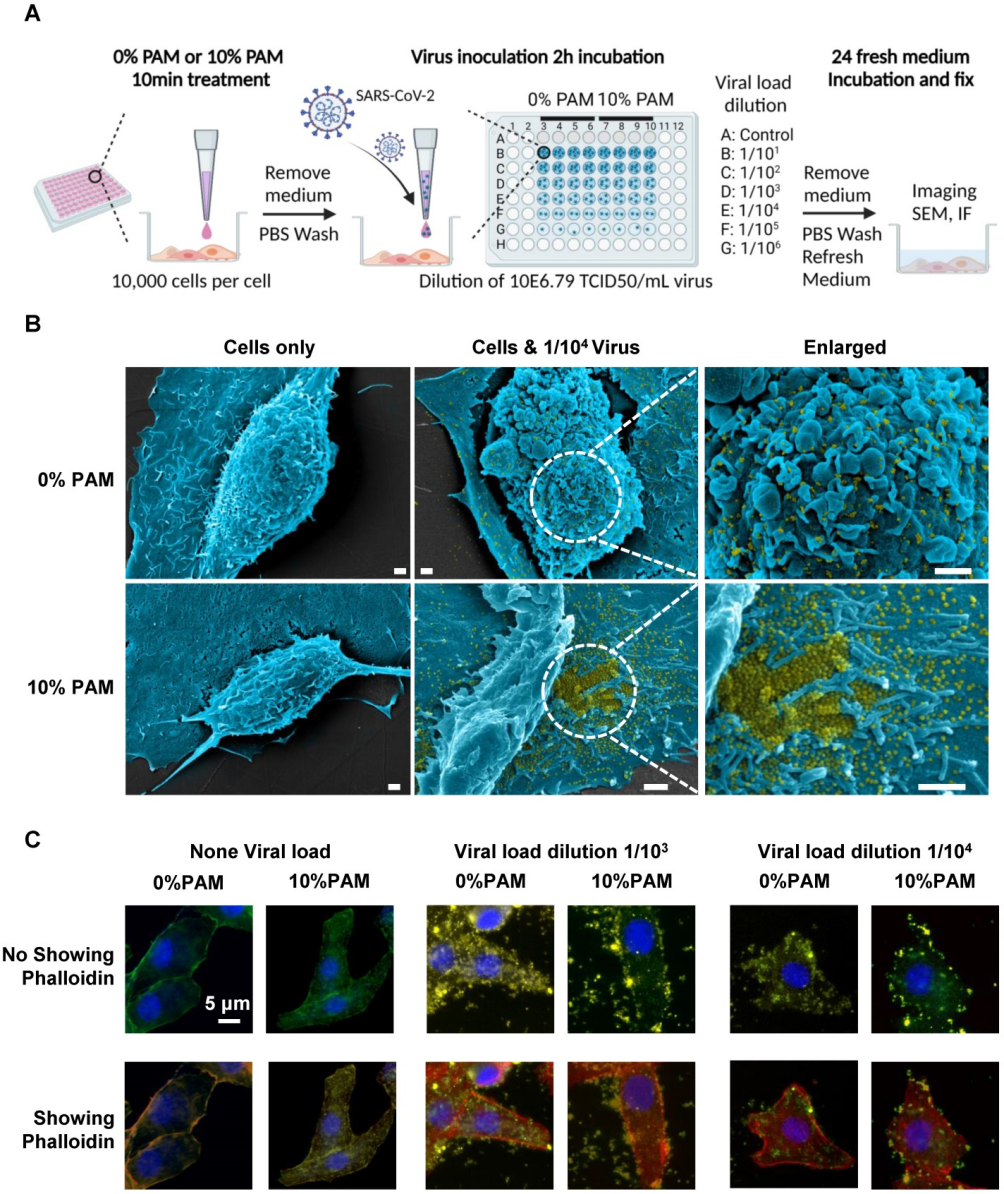
*Scheme of the CAP pre-treatment of cells for SARS-CoV-2 virus infection and the associated results.* (*A*) Conceptual scheme illustrating the protocol used in the SARS-CoV-2 virus experiments. Vero E6 cells were pre-treated with non-toxic concentrations of plasma-activated medium (PAM), 10% PAM or 0% PAM (as control) for 10 minutes prior to SARS-CoV-2-infection with dilutions of stock virus (10E6.79 TCID_50_/mL virus) as indicated. Cells were incubated with 10% FCS RPMI-1640 medium for 24 hours before fixation and assessment of cell number and area, imaging, immunofluorescence and SEM; (*B*) Scanning electron microscopy under different folds of magnification showing extracellular virus on Vero E6 cells that underwent 0% PAM or 10% PAM pre-treatment prior to the infection with 1/10^4^ dilution of 10E6.79 TCID50/mL SARS-CoV-2 virus. Extracellular viral particles are pseudocoloured in yellow; (*C*) Immunofluorescence for ACE2 (green) or SARS-CoV-2 viral particles (yellow), and staining for actin filaments (phalloidin; red) or DNA (Hoechst 33342; blue). Results were analyzed using the Incell 6500HS confocal high content screening microscope, and analyzed using the IN Carta Analysis software for automated unbiased analysis in a plane through the middle of the nucleus to assess staining within the cell or at the periphery.

We examined the efficacy of CAP in blocking SARS-CoV-2 cell entry using SARS-CoV-2 virus and 10% PAM pretreatment in the* in vivo* virus assay (Figure 2*A*). Vero E6 cells were treated once with 0% or 10% PAM for 10 min before addition of 10-fold serial dilutions of viruses. Cells started to show signs of cytopathic effects (CPE) in the 0% PAM group after 24h, while cells pre-treated with 10% PAM largely retained the normal morphology. As expected, the number and area of live cells increased as the virus was diluted (Figure S1A-B). Under SEM, extracellular viral particles (pseudocoloured yellow) were visible after 10% PAM treatment (Figure 2*B*), consistent with a preventive role of 10% PAM against SARS-CoV-2 invasion. Using the high-throughput Incell 6500HS confocal high content screening microscope, and IN Carta software for automated unbiased analysis in a plane through the middle of the nucleus, we also observed significant reductions in the amount of intracellular virus (p = 5.31E-4 for 1/10^3^, p = 1.54E-3 for 1/10^4^) and significant increases in virus at the periphery demarcated by phalloidin staining of actin (p = 4.08E-3 for 1/10^3^, p = 3.11E-3 for 1/10^4^) after 10% PAM treatment. Intracellular virus was almost entirely absent when virus titer was 1/10^4^ or less (Figure 2*C*, Figure S2A-B). We also observed that ACE2 was substantially internalized on 10% PAM treatment (p = 1.55E-3 for increased cytoplasmic ACE2, p = 2.17E-3 for decreased membrane ACE2) and that virus infection created a similar effect on translocating ACE2 from cell membrane to cytoplasm (taking 1/10^4^ as an example, p = 3.7E-3 for control, p = 8.7E-4 for 10% PAM) as what was seen with 10% PAM treatment (Figure 2C, Figure S2A-B).

Due to potential confounding by agent cytotoxicity in assessing CPE responses to viral infections, we ensured that the doses of PAM treatment assessed, and also direct CAP exposure as examined below, did not show any significant effect on the viability (Figure S3A-C) and apoptosis (Figure S3D-F) of MCF10A and Vero E6 cells. This also supports the safety of using CAP and PAM for SARS-CoV-2 infection prevention. Under the brief exposure conditions employed (5-10 min), the addition of FBS in the treatment medium did not alter cell cycle (Figure S4A). In order to rule out any cytostatic effects of CAP or PAM exposure, we treated cells with 100% PAM for 30 s or 1 min as used below in the *in vitro* experiments, which showed equivalent efficacy in halting cell growth under 10% PAM for 5-10 min or under 30% PAM for 1 min (Figure S3G). Cells underwent a slight increase in S phase with 5s CAP exposure or 5% PAM treatment, the enlarged S phase went back to normal with 10 s-30 s CAP exposure or 10%-30% PAM treatment. Cytostatic effects became obvious when the dose of CAP was increased to 60 s or above for direct exposure, or with 50% PAM treatment (Figure S4B-C). These results indicated that 10 s-30 s direct CAP exposure, 10% PAM treatment for 5-10 min, and 30% PAM treatment for 1 min had no detrimental effects on cell cycle and proliferation that could confound the CPE results and suggest that such doses and limiting time of exposure can potentially be safely applied in mammalian cells.

### Characterization and *in vitro* functionality assessment of pseudoviruses with ACE2-binding domain

**Figure 3:**
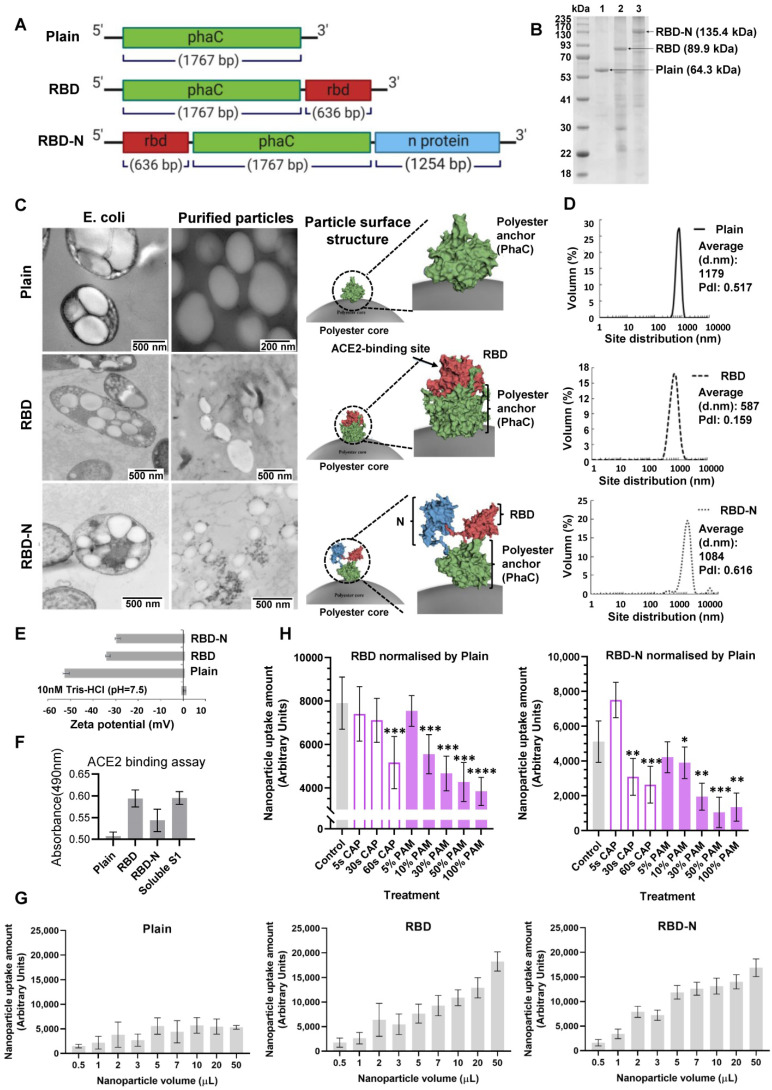
Design, bioengineering, characterization, and functional assessment of self-assembled ACE2-binding polyester particles, and the efficacy of CAP in blocking their entry into Vero E6 cells. (A) Schematic diagram of hybrid genes used for the production of ACE2-binding polyester particles (plain PhaC particles, PhaC-RBD particles, and RBD-PhaC-N protein particles); (B) Protein profile analysis of purified ACE2 binding polyester particles using 10% Bis-Tris gel electrophoresis. kDa, molecular weight marker (GangNam-Stain prestained protein ladder; iNtRon); lane 1, PhaC (64.3 kDa); lane 2, PhaC-RBD (89.9 kDa); lane 3, RBD-PhaC-N protein (135.4 kDa); (C) TEM images of purified ACE2 binding polyester particles and the corresponding ClearColi BL21(DE3) cells harboring these particles. (Scale bars: cells, 500 nm; purified particles, 200 nm or 500 nm). TEM images of PhaC-RBD particles, RBD-PhaC-N protein particles, and cells harbouring these particles. The depicted structural models of PhaC, PhaC-RBD, and RBD-PhaC-N protein were deduced from Phyre2 Protein Fold Recognition Server. The protein surface was rendered using Pymol. Green, the polyester anchor PhaC; red, RBD domain; blue, N protein; (D) Size distribution measurement of ACE2-binding polyester particles. The size distribution of each particle sample was consecutively measured three times. Each data point of measurement represents the mean ± the standard error of the mean. d.nm, diameter in nanometer. PdI, polydispersity index; (E) The ζ-potential of ACE2 binding polyester particles. The measurement of ζ-potential was consecutively measured three times. Each data point of measurement represents the mean ± the standard error of the mean; (F) Functionality assessment of ACE2-binding polyester particles, produced in an endotoxin free host ClearColi BL21(DE3), as per an ACE2-RBD binding assay; (G) Immunofluorescence intensities showing the entry amounts of PhaC particles, PhaC-RBD particles, and RBD-PhaC-N protein particles under different supplementing volumes; (H) Immunofluorescence intensities showing the entry amounts of PhaC-RBD and RBD-PhaC-N protein particles as normalized by PhaC particles under different CAP exposure approaches. The normalization was done following 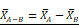, and 
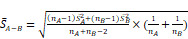
. The Y axis shows fluorescence intensity (arbitrary units).

We tested the preventive role of CAP against COVID-19 via fabricating ACE2-binding polyester particles. SARS-CoV-2 S1 glycoprotein contains the RBD domain, which directly binds to the peptidase domain (PD) of the host receptor ACE2^65^. We produced polyester particles that self-assembled inside engineered *E. coli*, and which were densely coated with RBD^58^. The ACE2-binding polyester particles were isolated and their utility as pseudovirus for the analysis of the protective role of CAP/PAM treatment against SARS-CoV-2 infection was investigated. Biological polyester particle formation in* E. coli* requires the introduction of three main enzymes, PhaA, PhaB, and PhaC. The first two enzymes provide the precursor molecules for PhaC-catalyzed polyester synthesis^62^. The PhaC enzyme remains covalently attached to the polyester particle surface after particle formation^61^,^62^. Thus, PhaC was used as an anchoring domain enabling oriented display of the RBD on the surface of polyester particles via translational fusion with PhaC. Hybrid genes were previously constructed to encode fusion proteins that mediated the *in vitro* self-assembly of ACE2-binding polyester particles displaying the ACE2-binding domain RBD and/or nucleocapsid N protein^58^. The modular composition of various hybrid genes and encoded recombinant proteins is illustrated in Figure 3*A*. Respective pET-14b plasmids containing hybrid genes were introduced into an endotoxin-free production host, ClearColi BL21(DE3) harboring pMCS69, containing the genes encoding enzymes PhaA and PhaB. The protein profile of ACE2 binding polyester particles (Figure 3*B*) was analyzed using 10% Bis-Tris gel electrophoresis to investigate whether the ACE2-binding RBD was covalently displayed on the particle surface. The full-length PhaC-ACE2-binding protein fusions were overproduced, and had been previously corroborated by identification of dominant fusion proteins with molecular weights (MWs) corresponding to the theoretical MWs of PhaC (64.3 kDa), PhaC-RBD (89.9 kDa), and RBD-PhaC-N protein (135.4 kDa), indicating that the RBD domain was successfully immobilized to the particle surface via the PhaC polyester anchor^58^. The target protein sequences of PhaC-RBD and RBD-PhaC-N protein had been identified by Q-TOF/MS^58^ (Table S1).

The *in vivo* formation of ACE2-binding polyester particles mediated by respective recombinant fusion proteins in ClearColi BL21(DE3) was also confirmed by TEM (Figure 3*C*). The 3D models of respective fusion proteins were deduced to illustrate the RBD structure on the polyester particle surface (Figure 3*C*). The majority of ACE2-binding polyester particles ranged between 300 nm and 1100 nm (Figure 3*D*). Particles with ζ-potential measurement higher than 30 mV or less than -30 mV were considered to have high degrees of stability^66^. The ζ-potential of ACE2-binding polyester particles was less than -30 mV (Figure 3*E*), suggesting that they possessed strong stability. In addition, the function of ACE2-binding polyester particles was assessed* in vitro* using an ACE2-RBD-binding assay (Figure 3*F*). The plain PhaC particle and free soluble S1 protein were used as negative and positive controls, respectively. The results (Figure 3*F*) demonstrated that the ACE2-binding polyester particles (PhaC-RBD and RBD-PhaC-N protein particles) exhibited a higher ACE2 binding when compared with the negative control, PhaC particle. The PhaC-RBD particle and RBD-PhaC-N protein particle showed similar ACE2 binding capacities when compared with the positive control, soluble S1. The binding results indicated that the ACE2-binding domain of RBD was functional, i.e., properly folded, when assembled into the surface of polyester particles inside engineered ClearColi BL21(DE3) corroborating our previous findings.

### CAP effectively prevents cells from pseudoviruses with ACE2-binding domain

The cell uptake rates of the polyester particles were dose-dependent and those coated with RBD or RBD-N fusion were taken up at considerably higher rates when compared with the control particles, suggesting that the presence of the ACE2-binding RBD mediates enhanced particle cell uptake. Carrying the ACE2-binding RBD domain or not did not alter the cell uptake rate if the applied particle volume was below 3 µL (Figure 3*G*), suggesting the existence of particle passive entry as a basic cell entry mode. Yet the rate reached a plateau at 5000 for the control particle, and the performance became stable when its dose reached 10 µL (Figure 3*G*). Thus, we used the 10 µL (0.1 mg/µL) volume of particles for further experiments.

CAP direct treatment for 60 s significantly reduced the uptake rate of both RBD (p = 0.034) and RBD-N (p = 0.039) protein particles (Figure 2*H)*. Incubating cells in 100% PAM (10 min-activated PAM), 50% PAM (100% PAM diluted to 50%), or 30% PAM (100% PAM diluted to 30%) for 1 min significantly inhibited the entry of particles coated with RBD or RBD-N (approximately 50% for RBD with p = 2E-4, and 30% for RBD-N with p = 3E-4, Figure 3*H*). Yet, 10% PAM (100% PAM diluted to 10%) could only effectively inhibit the uptake of particles coated with RBD (approximately 60% with p = 2E-4, Figure 3*H*).

### CAP suppresses ACE2 levels and triggers ACE2 translocation

**Figure 4:**
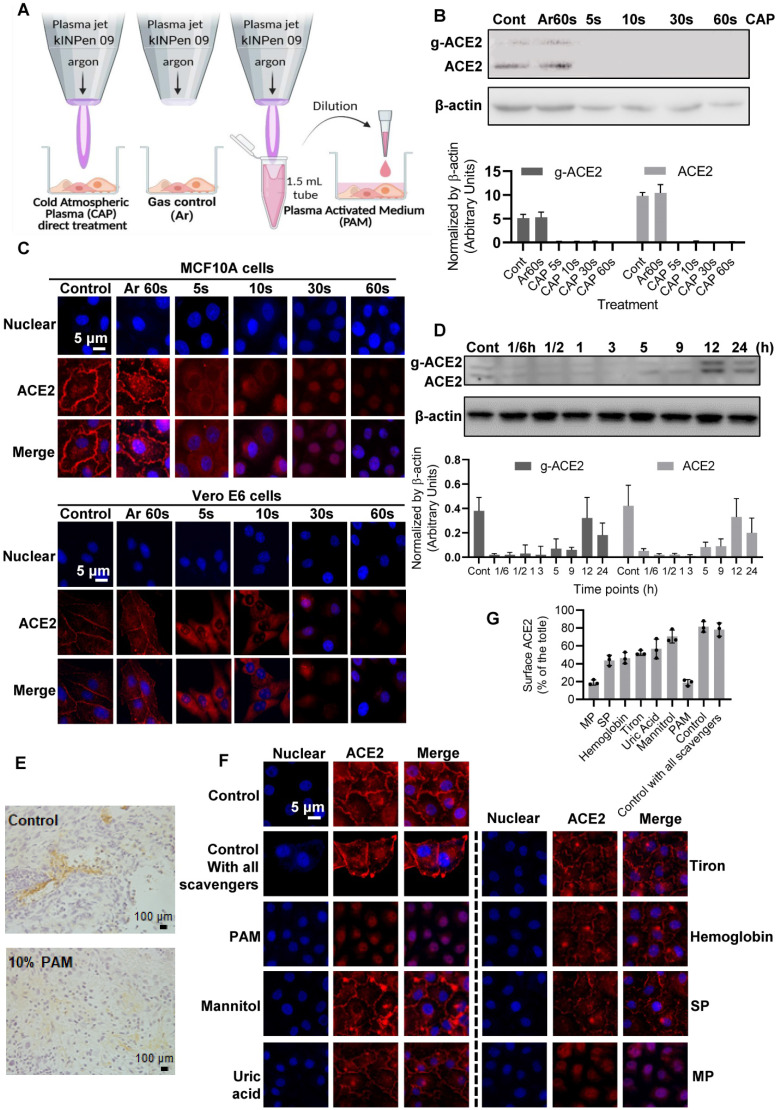
*ACE2 expression and location after PAM treatment and examination of the active CAP components triggering such an effect.* (*A*) Cells are treated directly with CAP or indirectly with PAM or gas control (Ar). (*B*) Western blot and quantification of ACE2 after direct CAP exposure as indicated. (*C*) Immunofluorescence images of ACE2 after direct CAP exposure in MCF10A and Vero E6 cells. (*D*) 30 s-PAM for 60 s treatment and wash with PBS. Refresh with 10% FBS RPMI-1640 medium for 1/6, 1/2, 1, 3, 5, 9, 12 and 24 h. The ACE2 protein expression levels were detected by Western blot. (*E*) IHC results from *in vivo* mouse experiments. Surface ACE2 expression as represented by glycolyzed ACE2 in cells with and without 10% PAM treatment using the SUM159PT-inoculated mice model. (*F*) Immunofluorescence images showing ACE2 localization and expression after direct CAP exposure for 30 s when different ROS scavengers were used, and signal quantification. In this assay, 200 mM mannitol, 100 μM uric acid, 20 mM tiron, 20 mM hemoglobin, 10 mM sodium pyruvate and 1 mM monopotassium were used to quench hydroxyl radical, ozone, superoxide anion, nitric oxide, H_2_O_2_, and e^-^, respectively. The second control in (*A*) and (*B*) was '60 s Argon (Ar) exposure'. The dosing effect of CAP was measured under different treatment durations in the form of plasma activated medium (PAM). MCF10A cells were used. 'g-ACE2' is short for 'glycosylated ACE2' and 'ACE2' represents 'non-glycosylated ACE2'. Scale bar: 5 μm. (*G*) The quanlification data of (*F*).

Considerable reduction in the intensity of bands consistent with glycosylated and non-glycosylated forms of ACE2 was observed after CAP treatment in MCF10A cells (Figure 4). Compared with the control and Ar treatment (Gas control) (Figure 4A), the inhibitory effect of direct CAP treatment was visible even at only 5 s CAP exposure (Figure 4*B*). A 90% ACE2 reduction was observed when the exposure duration was sustained to 60 s for both MCF10A and Vero cells (Figure 4*C*). Translocation of ACE2 to the nucleus was evident by immunofluorescence after 30 s direct CAP exposure (Figure 4*C*). Similar results were obtained using 30 s-CAP-activated medium (30 s-PAM) and water (30 s-PAW) (Figure S5*A*). Significant reduction of ACE2 levels was achieved when cells were incubated with PAM for 5 s and nuclear translocation of ACE2 was evident after incubation with PAM for 30 s. ACE2 became almost invisible when the incubation duration increased to 60 s (Figure S5B-C). The inhibitory effect of PAM (30 s-PAM treatment for 60 s) on ACE2 levels lasted for 5 h, with a return to the initial level by 12 h (Figure 4*D*). Significantly reduced expression of glycosylated ACE2 was observed after receiving 10% PAM treatment in an *in vivo* mouse model inoculated with SUM159PT cells (Figure 4*E*). A tumor model was used to easily track cells of human origin.

### Hydroxyl radical and its derivatives play the leading role in triggering ACE2 nucleus translocation

Adding mannitol (·OH scavenger) led to significant reversal of ACE2 cell surface distribution (p = 1.79E-4, Figure 4*F*) that is comparable with the negative control (*i.e.*, adding DMEM, p = 0.058, Figure 4*F*), suggesting the leading role of hydroxyl radical and its derivatives generated via interacting with other reactive species in triggering ACE2 nucleus translocation. Supplementing cells with uric acid (O_3_ scavenger), tiron (∙O_2_^-^ scavenger), hemoglobin (∙NO scavenger) and sodium pyruvate (H_2_O_2_ scavenger), each partially reversed the effect of PAM on ACE2, yet adding monopotassium (e^-^ scavenger) did not convey any visible effect (Figure 4*F*). Supplementing cells with all scavengers without PAM treatment did not alter ACE2 expression or localization (Figure 4*F*), suggestive of the neglectable cellular basal ROS level on ACE2.

### CAP triggers translocation of ACE2 to the nucleus via the ER/STAT3 axis

**Figure 5:**
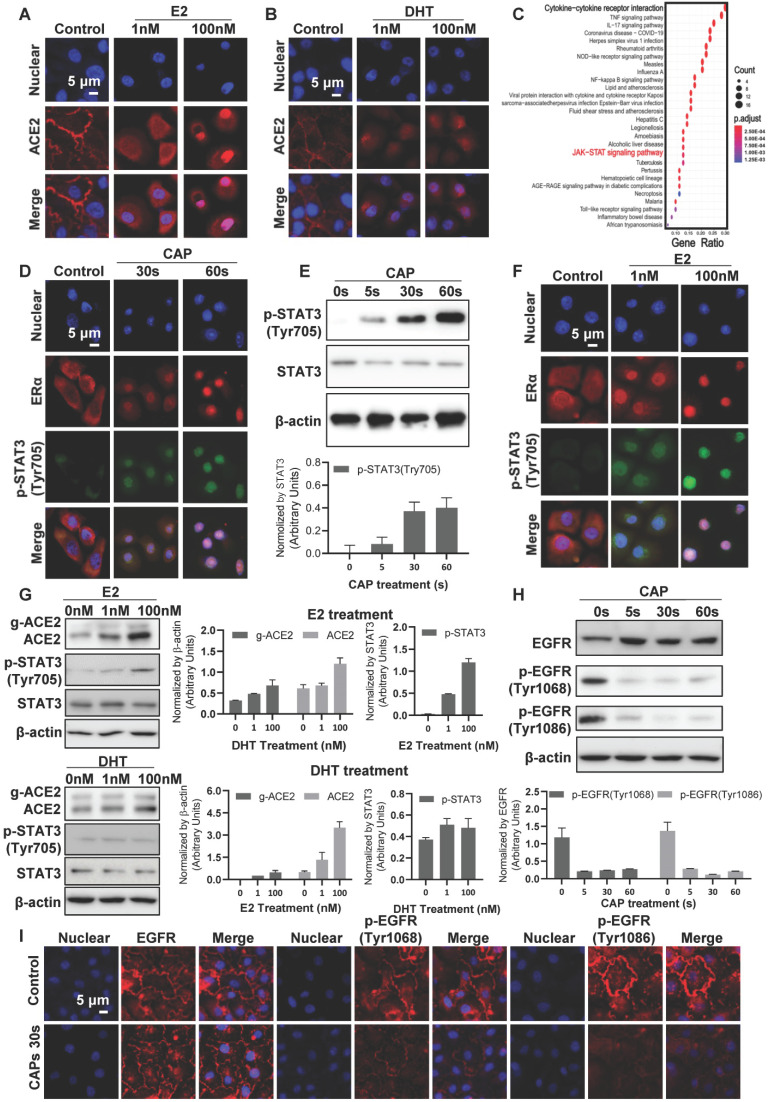
*Cell signaling after direct CAP exposure for different durations.* Immunofluorescence images showing ACE2 localization and expression in response to (*A*) E2, and (*B*) DHT. (*C*) KEGG pathways enriched by differentially expressed genes in human bronchial epithelial (NHBE) cells on SARS-CoV-2 infection. (*D*) Immunofluorescence images showing ERα and p-STAT3(Tyr705) location and expression after direct CAP exposure for different durations as indicated. (*E*) Western blot and quantification showing cell surface ACE2 (g-ACE2) ACE2, p-STAT3(Tyr705), STAT3 expression after direct CAP exposure for different durations. (*F*) Immunofluorescence images showing ERα and p-STAT3(Tyr705) location and expression in response to E2. (*G*) Western blot and quantification showing cell surface ACE2 (g-ACE2) ACE2, p-STAT3(Tyr705), STAT3 expression in response to E2 and DHT, respectively. MCF10A cells were used. 'g-ACE2' is short for 'glycosylated ACE2' and 'ACE2' represents 'non-glycosylated ACE2'. Scale bar: 5 μm. (*H*) Western blot and quantification showing EGFR and p-EGFR(Tyr1068/1086) expression after direct CAP exposure for different durations (0 s, 5 s, 30 s and 60 s). (*I*) Immunofluorescence images showing EGFR and p-EGFR(Tyr1068/1086) on 30 s direct CAP exposure.

Cell surface ACE2 was internalized on CAP exposure and the effect increased with the duration and dose (defined as the exposure duration of medium to CAP during PAM preparation) of CAP/PAM/PAW used for cell treatment (Figure 4*B*-*D*, Figure S5). Interestingly, similar effects were observed when cells were treated with the female (estrogen; E2) but not the male (di-hydro-testosterone; DHT) hormone (Figure 5*A*-*B*). To explore the potential underlying mechanism, we identified differentially expressed genes on SARS-CoV-2 infection in human bronchial epithelial (NHBE) cells and mapped them to KEGG pathways using GSE147507^67^ from the Gene Expression Omnibus (GEO) database. Among the top 25 pathways enriched by the 105 differentially expressed genes in response to SARS-CoV-2 infection (Figure 5*C*, Table S2), 'cytokine-cytokine receptor interaction' was ranked as the top, and JAK/STAT3 signaling, the pathway that mediates cytokine signaling^68^ was identified as up-regulated upon virus infection (Figure 5C, Figure S6A). JAK/STAT signaling was recently proposed to be targeted for cytokine release syndrome control in COVID-19^69^. We, therefore, explored the role of JAK/STAT signaling in CAP-triggered ACE2 internalization.

STAT3 (Tyr705) phosphorylation was low and primarily localized in cytoplasm under control conditions (Figure 5D, Figure S6B, D). CAP exposure activated STAT3 (Tyr705) phosphorylation and translocated phosphorylated STAT3 (Tyr705) into the nucleus, which became more evident with the exposure duration of cells to direct or indirect CAP treatment (Figure 5D, Figure S6). Specifically, elevated STAT3 phosphorylation was clearly evident after direct CAP exposure or 30 s-activated PAM/PAW incubation, starting from 5 s (Figure 5E, Figure S6C, E, S7A-B). Phosphorylated STAT3 nuclear translocation was visible when the exposure duration increased to 30 s, and became obvious at 60 s (Figure 5*D*, Figure S6B-E).

Estrogen receptor α (ERα) binds the promoter region of STAT3 (core score =1), and STAT3 transcriptionally regulates ACE2 expression (core score =1) according to computational predictions using ConTra (V3)^70^, suggesting that STAT3 phosphorylation is positively associated with ACE2 expression and regulated by E2/ERα. Immunofluorescence revealed ERα to be evenly distributed in cells including cell surface, cytoplasm and nucleus under control conditions, and co-localized with phosphorylated STAT3 (Tyr705) on CAP exposure (Figure 5*D*, *F*). Nuclear translocation of ERα and phosphorylated STAT3(Tyr705) was initiated after 30 s direct CAP treatment, and almost all ERα and p-STAT3(Tyr705) were co-localized in the nuclei after 60 s direct CAP exposure (Figure 5*D*). Similar effects were observed for 30 s-activated PAM and PAW (Figure S6B, D). The same dose-dependent ERα-STAT3(Tyr705) nuclear co-translocation was observed in cells treated with E2 (Figure 5*F*). The physiological level of E2 (1 nM) created a similar efficacy as 5 s-activated PAM in triggering ERα signaling, and the response was dependent on relative endogenous ERα receptor functionality in cells as reflected in expression of the ERα target gene TFF1 (*i.e.,* strongest in MCF-7, intermediate in MCF10A, and not seen in MDA-MB-231 cell lines) (Figure S7C). While E2 caused activation of ACE2 and p-STAT3(Tyr705), DHT did not cause any effect (Figure 5*G*). These suggested that ACE2 internalization was specifically mediated via the ERα/STAT3 axis.

### CAP suppresses ACE2 expression via activating EGFR/STAT3 signaling

Besides nuclear internalization, ACE2 expression decreased with the dose of CAP/PAM/PAW (Figure 4*B*-*D*, Figure S5). As EGFR-dependent STAT3 signaling plays critical roles in gene regulation^71^, and p-EGFR(Tyr1068) is the primary docking site for STAT3^72^, we analyzed the expression and localization profiles of both EGFR and STAT3 under different CAP exposure durations. While CAP up-regulated the level of p-STAT3(Tyr705) in a dose-dependent manner, it substantially blocked EGFR phosphorylation at Tyr1068 and Tyr1086 sites (Figure 5*H*, *I*, Figure S8), suggestive of decreased expression of downstream genes regulated by EGFR/STAT3 and that the regulation of STAT3 on ACE2 relies on EGFR(Tyr1068/1086) activation.

## Discussion

We found from this study that both direct CAP exposure and indirect PAM/PAW pre-treatment of cells could prevent them from SARS-CoV-2 infection via triggering ACE2 internalization using both designed pseudovirus particles and SARS-CoV-2 viruses. Remarkably, estradiol proved similar to CAP in causing ACE2 translocation from cell surface to the nucleus, whereas dihydrotestosterone did not. This, on the one hand, is consistent with the epidemiological evidence that females are less likely to develop severe COVID-19 than males^73^ and, on the other hand, provides additional support on the role of ACE2 internalization in protecting cells from SARS-CoV-2 infection.

The effect of PAM on ACE2 is dose-dependent as reflected by the increased uptake of bioengineered pseudoviruses carrying ACE2-binding spike protein domains with the increased CAP exposure duration or PAM concentration (Figure 3). The recommended safe dose for use, *i.e.*, 30% PAM for 1 min treatment or 10% PAM for 5-10 min treatment (Figure S4G), works for both pseudo and real SARS-CoV-2. The inhibitory effect of 30s PAM (30s CAP-activated PAM) or 30% PAM (10 min CAP-activated PAM diluted to 30%) on SARS-CoV-2 infection after 1-min exposure can last 5-12 h (Figure 4D, Figure S5B-C), suggestive of its consistent role in being used for regular COVID-19 epidemic prevention.

Unlike SARS-CoV-2 viruses, the pseudoviruses produced using polyester particles have a passive entry mode that enables their basic cell entry. This level reached a plateau at 5 to 10 µL, and particles carrying the ACE2-binding RBD domain alone or together with the N domain showed increasing uptake patterns, confirming the functionality of the ACE2-binding RBD attached to the polyester core of the particles and its suitability in mimicking the cell entry process of SARS-CoV-2. In addition, the RBD-coated particles have a higher entry capacity than the RBD-N protein coated particles, which also carry an N domain, suggesting a reduced entry with the increase of the particle size as a result of the additional protein domain. The positive relationship between PAM concentration and the size of entry complexes it can inhibit (Figure 3H) further consolidated its dose-dependent efficacy.

It has been proposed that cool temperatures (12 to 19°C) reduce the endocytosis of virus-receptor complexes, and a combinatorial use of cool air therapy (CAT) and purified air technology has been proposed to reduce SARS-CoV-2 load and COVID-19 severity^74^. Our experiments were conducted at the room temperature that ranges from 15 to 20°C, suggestive of the validity of the proposed CAP-based preventive strategy against SARS-CoV-2 infection. This is because the recessed receptor endocytosis as a result of decreased membrane fluidity may hold true without external perturbation, but can be violated on PAM treatment due to altered cellular redox fluctuations and cell response adjustment. On the other hand, the influence of temperature on the binding affinity of SARS-CoV-2 to host receptors and the subsequent endocytosis may be limited as COVID-19 spreads throughout all seasons.

We have critically assessed the safety of CAP/PAM on cells considering that cells represent the smallest and final unit that receive external signals and generate response, and CAP/PAM doses used acceptable at the cell level should be nontoxic at the levels of tissues, organs and the whole body. Specifically, the recommended CAP/PAM dose for use (30s or 30% PAM for 1 min, or 10% PAM for 5-10 min) doesn't convey any cytotoxicity (as examined by cell viability and apoptosis) or negative effect on cell cycle (Figure S4-5). Beyond this, we have previously assessed the CAP/PAM safety at the animal level as a potential onco-therapy^75^. In that study, percentages of various blood cells such as monocytes, neutrophil granulocyte, eosinophil granulocyte, and basophil granulocyte were restored back to normal after CAP/PAM treatment without any adverse effect, and the dose of CAP/PAM used for* in vivo* cancer treatment was higher than what was used in this study, further supporting its safe use as a possible preventive strategy against COVID-19.

We used SARS-CoV-2 virus as the *in vivo* model and self-designed pseudovirus as the* in vitro* model here to examine the effect of CAP/PAM on ACE2 and SARS-CoV-2 intake. Unlike other pathogenic conditions such as cancers, where mice models are canonically used to validate *in vitro* findings, SARS-CoV-2 cannot infect wild-type laboratory mice due to inefficient interactions between the viral spike protein and the mouse ortholog of ACE2^76^. A mouse-adapted virus model of SARS-CoV-2 (SARS-CoV-2 MA) was established via reverse genetics to remodel interactions between SARS-CoV-2 spike protein and mouse ACE2 which, though capable of infecting mice, could not grow as efficiently as the wildtype SARS-CoV-2 in the primary human bronchiolar epithelial cells and Vero cells^77^. Thus, SARS-CoV-2 MA could be considered as an artificial SARS-CoV-2 variant favoring mice infection at a sacrifice of the fitness in infecting human cells, which is against the evolutionary trend of SARS-CoV-2 and not an ideal model for this study. Alternatively, human ACE2 (hACE2) was stably expressed in mice under the control of mouse ACE2 (mACE2) promoter using the CRISPR/Cas9 technique to create a mouse model (termed hACE2-KI/NIFDC mice) susceptible to SARS-CoV-2 infection. However, unexpected results such as the presence of viral RNAs in the mice brain were observed that do not exist among COVID-19 patients^78^, and no clinical symptom or mortality was observed in hACE2-KI/NIFDC mice that warrants its use in recapitulating severe COVID-19^79^. Large animal models for SARS-CoV-2 infection such as rhesus macaque^80^,^81^, cynomolgus macaque^82^ and African green monkey^83^ are advantageous in having similar pathology with human and immune responses that resemble COVID-19 patients without genetic modifications. However, these models have limited availability, ethical concerns, and difficulties in reaching appropriately powered group sizes despite their high cost^84^.

Also worth emphasizing is that the leading role identified for ·OH is not limited to its single effort but also includes reactive species produced through its interactions with other species. Due to the extreme short free diffusion path length (approximately 5 nm) and short half-life span (around 1 ns) of ·OH^85^, it may not be able to reach cell surfaces. Instead, ·OH can form H_2_O_2_ (i.e., via ·OH→H_2_O_2_) or singlet oxygen (O_2_^1^) (i.e., via 4·OH→2H_2_O+O_2_^186^ that may alter cell surface including what we observed on ACE2, given their substantially enhanced stabilities (i.e., diffuse path length: ~250 nm for O_2_^1^, ~1 cm for H_2_O_2_; half-life span: ~1 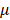
s for O_2_^1^, ~20 s for H_2_O_2_). Below we use a tumor model as an example to illustrate the possible effect of O_2_^1^ and H_2_O_2_ on cells. Singlet oxygen could cause local inactivation of a few catalase molecules on the surface of cancer cells that leads to H_2_O_2_ influx via aquaporins and ultimately tumor cell death, and the accumulation of H_2_O_2_ and ONOO^-^ at the site of locally inactivated catalase towards self-sustained autoamplification of O_2_^1^ generation and catalase inactivation^87^.

**Figure 6:**
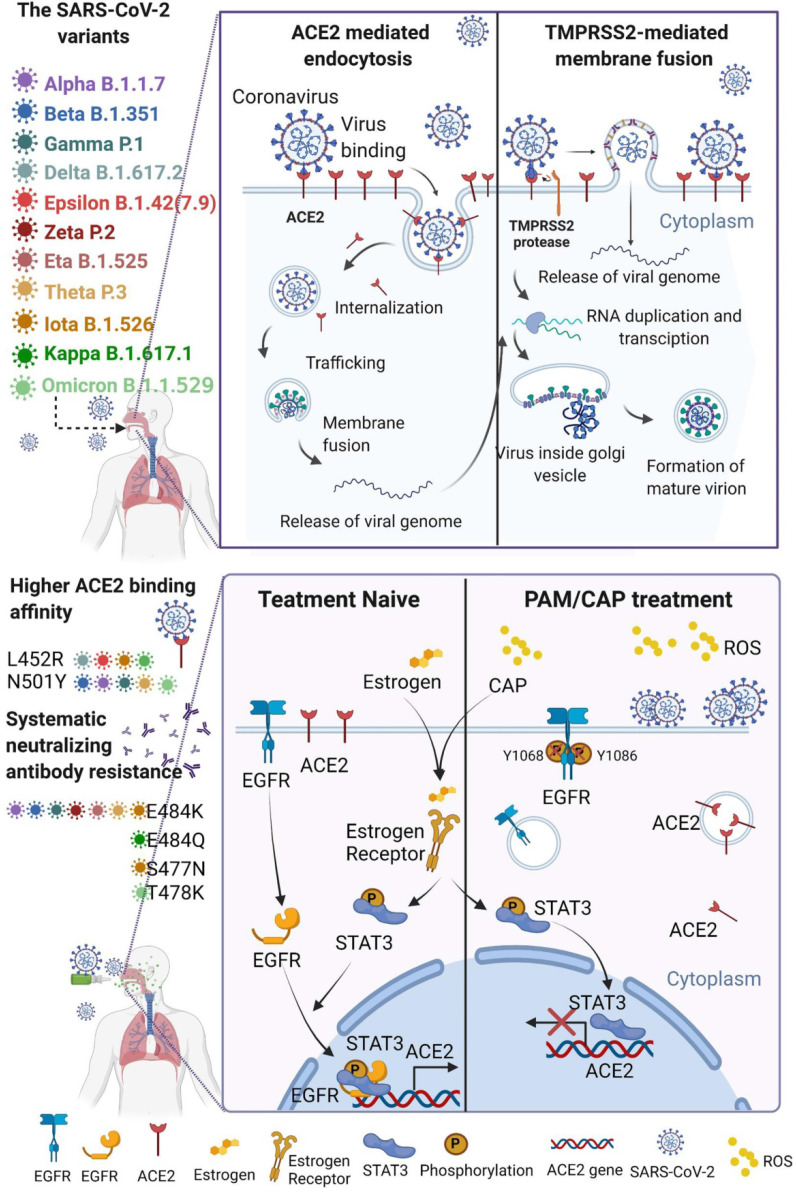
*Schematic representation of virus entry into host cells and plasma protection mechanism.* SARS-CoV-2 and its mutated variants enter host cells by receptor-mediated endocytosis and/or TMPRSS2-mediated membrane fusion, which both rely on ACE2. The variants have higher affinity to ACE2 and are resistant to systemic neutralizing antibody. Both CAP or PAM and estrogen regulate ACE2 expression and cause ACE2 nuclear translocation that involves ERα/STAT3 signaling. Without ACE2 cell surface expression, SARS-CoV-2 virus loses entry and is blocked on the cell surface. CAP activates ERα that is translocated into the nucleus and contributes to enhanced ACE2 expression through the ER/STAT3(Tyr705)/EGFR(Tyr1068/1086) axis. When CAP exceeds a certain level, it suppresses EGFR(Tyr1068/1086), which blocks EGFR-dependent STAT3 signaling and consequently reduces ACE2 expression. MCF10A cells were used. Schematic figure created with BioRender.com (Access date: 10^th^ June, 2021).

ACE2 is not the sole membrane receptor affected by CAP/PAM that involves, at least, ERα and EGFR, among others. Importantly, interactions between the ERα/STAT3 and EGFR/STAT3 may collectively determine the localization and levels of ACE2. We have three observed facts on CAP: 1) enhancing ACE2 internalization (Figure 4C); 2) activating ERα nucleus translocation that transcriptionally activates STAT3 and enhances STAT3(Tyr705) phosphorylation (Figure 5D); 3) suppressing EGFR(Tyr1068/1086) phosphorylation (Figure 5I). Phosphorylated EGFR and STAT3 are known to synergize in promoting the expression of downstream genes such as STAT1^88^ and, thus, possibly ACE2 as well that leads to suppressed ACE2 expression but enhanced internalization in response to CAP. Estrogen conveys similar effects with CAP by activating ERα, STAT3 and ACE2 nucleus translocation. Collectively, ACE2 expression largely relies on ERα/STAT3 and EGFR/STAT3 signaling. While activated ERα, either by CAP or estrogen, enhances ACE2 expression via activating STAT3 (the ERα/STAT3 axis, Figure 6), CAP overdose suppresses ACE2 expression by suppressing EGFR(Tyr1068/1086), whereas the synergy between EGFR(Tyr1068/1086) and STAT3(Tyr705) is crucial for activating ACE2 expression (the EGFR/STAT3 axis, Figure 6). This possibility requires further experimental validations as well as tests using preclinical and clinical models.

Interestingly, SARS-CoV-2 infection may create a similar efficacy in ACE2 internalization as CAP. SARS-CoV was known to cause ACE2 internalization 89,90, and a similar mechanism has been proposed for SARS-CoV-2 infection^91^. Importantly, SARS-CoV-2 was reported to stimulate the innate immune response by triggering, among others, ROS generation^92^,^93^. Thus, ACE2 nuclear translocation may be part of a natural self-protection process in response to increased cellular redox level given the role of ACE2 in maintaining cell redox homeostasis^94^. Besides, the JAK/STAT pathway was significantly up-regulated on SARS-CoV-2 infection (Figure 5*C*) and JAK/STAT3 signaling was activated on CAP exposure in healthy cells (Figure 5*D*-*E*). Thus, we could consider CAP potentially as a complementary innate immune-stimulatory treatment against SARS-CoV-2 infection in the sense that it has no virulence but is capable of creating a redox environment resembling virus infection^95^,^96^. Such non-specific type of immune boosting is of particular relevance to diseases caused by RNA viruses such as SARS-CoV-2 that undergo rapid evolution and mutation and may complement the current vaccination portfolio to achieve ultimate COVID-19 control. According to the estimating model reported on 25 May 2021^97^, symptomatic infections and critically ill patients have the same virus transmission ability. Although vaccines can now control severe symptoms, preventing virus spread is still the main approach for controlling COVID-19 epidemics. In addition to the enhanced binding affinity with ACE2, variants such as the Delta and Lambda mutant viruses carry multiple mutations in the Spike protein S1 subunit that help it escape from immune surveillance and furin cleavage^11^,^98^. This makes the protective effect of CAP against SARS-CoV-2 infection even more encouraging given its demonstrated roles in boosting the immune responses^99^,^100^.

Importantly, several reactive species have been proven to activate the innate immunity response in the initial stage of viral infection. Hydrogen peroxide (H_2_O_2_), hydroxyl free radical (·OH) and nitric oxide (NO) can cause innate immune activation similar to that caused by viral entry, via stimulating the TLRs/NF-κB/TNF-α pathway^101^. Therefore, the hypothesis that hydrogen peroxide spray on nasal, mouth and throat could prevent viral infection was proposed 102, based on which, a clinical trial (NCT04878042) was initiated on May 7, 2020. Besides the immune-regulatory role of reactive species, NO was demonstrated to be capable of directly s-nitrosylating viral proteins^103^ and thus had been regarded as an antiviral therapy^104^. A phase II clinical trial (NCT04337918) examining the efficacy of nasal spray of nitric oxide releasing solution (NORS) had been completed. Successful completion of these two clinical trials could provide solid support of the clinical application of PAM. In addition, unlike these two trials where single formulas of reactive species were tested, PAM is a multi-component solution, which not only conveys efficacies of these two particles simultaneously but also consecutively produces short-lived species through secondary reactions. This renders the clinical translation of PAM as an effective antiviral modality more promising.

CAP and its liquid derivatives (PAM/PAW) represent a chemically mild yet effective approach for COVID-19 protection through re-locating ACE2 away from cell surface. Similar to sunscreen, frequent use of PAM/PAW could be recommended to prevent the loss-of-effect on internalizing ACE2. PAM/PAW could be developed into products such as nose/mouth spray, eye drops, and handwash, etc., to aid in COVID-19 prevention among healthy individuals. Taking nose/mouth spray as an example, it may contribute to regular epidemic prevention without a need of daily mask wearing. Extension to oral cavity and respiratory tract may be possible with further delivery modalities, trials and regulatory approvals. It is worth highlighting the significant role of short-lived species in PAM that could not be simplified as long-lived species such as H_2_O_2_. This presents us with the challenge of preserving the activity of PAM if developed into products for COVID-19 prevention. One possibility is to explore potential CAP activity preservatives such as hyaluronic acid, which is considered a stabilizer of many active components^105^. Alternatively, we may take advantage of the secondary reactions that could convert long-lived into short-lived species (H_2_O_2_ →·OH + ·OH, O_3_ + H_2_O → 2·OH + O_2_, O_3_ + HO_2_ → ·OH + 2O_2_)^51^,^106^, and explore the possibility of adding corresponding catalyzers before use.

Besides functioning as a receptor interacting with SARS-CoV-2, ACE2 plays beneficial roles in the cardiovascular system via modulating the renin-angiotensin system (RAS)^91^,^107^. ACE2 negatively regulates the RAS via counteracting ACE, an angiotensin-converting enzyme that mediates angiotensin II production to cause cardiovasculare diseases especially hypertension^108^. Unlike SARS-CoV-2 triggered ACE2 internalization where ACE2 is occupied to form the early endosome that enables virus endocytosis in a clathrin or lipid raft-dependent pathway^91^, CAP-induced ACE2 internalization may increase its availability in taking on other functionalities by sparing it away from cell membrane. However, CAP reduced the level of ACE2 that may, on the other hand, not adversely affect its enzymic activity (similar to ACEI that differs in its effects on ACE2 expression and activity^109^). Thus, whether CAP-based COVID-19 strategy is feasible for long-term use or people carrying cardiovascular disorders without health issues requires additional experimental and clinical investigations. Nevertheless, the use of CAP as a short-term or intermittent prevention approach against COVID-19 is clinically relevant, as well as its application as an adjuvant therapy in, e.g., rescuing the life of an acutely infected patient by stopping virus spread and restricting infected area.

In conclusion, direct CAP or indirect PAM/PAW could be used to prevent SARS-CoV-2 infection by removing ACE2 from the surface of cells, in a CAP dose-dependent manner. This was achieved mainly through hydroxyl radicals and reactive species it generated through interacting with other species via activating ERα/STAT3 and EGFR/STAT3 signaling. We recommend exposing mucosal cells to CAP for 30s or 30% PAM for 1 min as a preventative that could reduce SARS-CoV-2 infection for 5-12 h. Specifically, nose/mouth spray and other products in the form of aerosols may be effective in a therapeutic setting, and handwash made of PAM may also help prevent SARS-CoV-2 infection in a sense that PAM could disable virus binding by damaging the RBD spike^110^ in addition to the reported efficacy on mucosal host cells here^111^. The epidemiologic observation that females are less likely to develop severe COVID-19 could partially be explained by the finding that the female sex hormone E2 resembles CAP in triggering ACE2 nuclear translocation and, importantly, is supportive to the protective role of ACE2 internalization in fighting against severe SARS-CoV-2 infection. The prevention of CAP against viral diseases is not limited to SARS-CoV-2 but also applicable to any other viruses utilizing ACE2 for cell entry, and aids in the control of a novel pandemic situation caused by viruses targeting host ACE2 in the future.

## Supplementary Material

Supplementary figures and tables.

## Figures and Tables

**Table 1:** Bacterial strains and plasmids used in this study.

Strains/plasmids	Characteristics/DNA sequences	References
*E. coli*		
*E. coli* Top10	F^-^ mcrA Δ(mrr-hsdRMS-mcrBC) φ80lacZΔM15 ΔlacX74 recA1 araD139 Δ(araleu)7697 galU galK rpsL (StrR) endA1 nupG	Invitrogen
*ClearColi* BL21(DE3)	F^-^ *ompT hsdS_B_ (r_B_^-^ m_B_^-^) gal dcm lon* λ (DE3 [*lacI lac*UV5-T7 gene 1 *ind1 sam7 nin5*]) *msbA148* ∆*gutQ* ∆*kdsD* ∆*lpxL* ∆*lpxM ∆pagP* ∆*lpxP* ∆*eptA*	Lucigen
Plasmids		
pUC57 n protein	Ap^R^, ColE1 origin, cloning vector containing *Xho*I/*Bam*HI fragment gene *n protein*	Biomatik
pUC57 rbd 5' end	Ap^R^, ColE1 origin, cloning vector containing *Xha*I/*Not*I fragment gene *rbd*	Biomatik
pUC57 rbd 3' end	Ap^R^, ColE1 origin, cloning vector containing *Xho*I/*Bam*HI fragment gene *rbd*	Biomatik
pET-14b phaC	pET-14b containing *phaC* DNA fragment; Ap^R^; T7 promoter	57
pET-14b phaC-rbd	pET-14b containing *Xho*I/*Bam*HI fragment gene *rbd* fused to the 3' end of *phaC*	58
pET-14b rbd-phaC-n protein	pET-14b containing *Xba*I/*Not*I fragment gene *rbd* and *Xho*I/*Bam*HI fragment gene *n protein* fused to the 5' end 3' end of *phaC respectively*	58
pMCS69	Cm^R^, pBBR1 MCS derivative containing *phaA* and *phaB* from *Cupriavidus necator*, colinear to lac promoter	59

Ap^R^, ampicillin resistance; Cm^R^, chloramphenicol resistance.
