# Supplementary material for: Cold atmospheric plasma for preventing infection of viruses that use ACE2 for entry
Source: Theranostics. 2022 Mar 14;12(6):2811–32. doi: 10.7150/thno.70098 (PMC8965494; doi:10.7150/thno.70098)
Supplement: Supplementary file 1 — Supplementary figures and tables. [file thnov12p2811s1.pdf]

Cold atmospheric plasma for preventing infection of viruses that use ACE2 for entry

Peiyu Wang[a,b,c,d,1], Renwu Zhou[e,f,1], Rusen Zhou[e,f,1], Wenshao Li[g], Janith Weerasinghe[e], Shuxiong Chen[h], Bernd H. A. Rehm[h], Liqian Zhao[i], Francesca D. Frentiu[c,j], Zhifa Zhang[a], Kexin Yan[k], Mary Lor[k], Andreas Suhrbier[k,l], Derek Richard[c,d], Erik W. Thompson[c,d], Kostya (Ken) Ostrikov[e], and Xiaofeng Dai[a,1,2]

[a]Wuxi School of Medicine, Jiangnan University, Wuxi 214122, China

[b]State Key Laboratory of Molecular Vaccinology and Molecular Diagnostics & Center for Molecular Imaging and Translational Medicine, School of Public Health, Xiamen University, Xiamen 361102, China.

[c]School of Biomedical Sciences, Queensland University of Technology, Brisbane 4059, Australia

[d]Translational Research Institute, Woolloongabba, Queensland 4102, Australia

[e]School of Chemistry and Physics, Queensland University of Technology, Brisbane 4000, Australia

[f]School of Chemical and Biomolecular Engineering, University of Sydney, Sydney, Australia

[g]School of Biology and Environmental Science, Queensland University of Technology, Brisbane 4000, Queensland, Australia

[h]Centre for Cell Factories and Biopolymers, Griffith Institute for Drug Discovery, Griffith University, Nathan, QLD 4111, Australia

[i]The First School of Clinical Medicine, Southern Medical University, Guangzhou 510515, China

[j]Centre for Immunology and Infection Control, Queensland University of Technology, Brisbane 4059, Australia

[k]QIMR Berghofer Medical Research Institute, Herston QLD 4006, Australia

[l]Australian Infectious Disease Research Centre, GVN Center of Excellence, Brisbane, Queensland, Australia.

[1]These authors contributed equally to this work.

[2]To whom correspondence may be addressed. Email: xiaofeng.dai@jiangnan.edu.cn

Table S1. Q-TOF/MS analysis of PhaC-SARS-CoV-2 antigen fusion proteins.

Protein/Protein sequence*	Peptide fragments assigned to the various protein regions
PhaC-RBD (89.9 kDa)	
MATGKGAAASTQEGKSQPFKVTPGPFDPATWLEWSRQWQGTEGNGHA<br>AASGIPGLDALAGVKIAPAQLGDIQQRYMKDFSALWQAMAEGKAEATG<br>PLHDRRFAGDAWRTNLPYRFAAAFYLLNARALTELADAVEADAKTRQR<br>IRFAISQWVDAMSPANFLATNPEAQRLLIESGGESLRAGVRNMMEDLTR<br>GKISQTDESAFEVGRNVAVTEGAVVFENEYFQLLQYKPLTDKVHARPLL<br>MVPPCINKYYILDLQPESSLVRHVVEQGHTVFLVSWRNPDASMAGSTWD<br>DYIEHAAIRAIEVARDISGQDKINVLGFCVGGTIVSTALAVLAARGEHPAA<br>SVTLLTTLLDFADTGILDVFVDEGHVQLREATLGGGAGAPCALLRGLEL<br>ANTFSFLRPNDLVWNYVVDNYLKGNTPVPFDLLFWNGDATNLPGPWYC<br>WYLRHTYLQNELKVPGKLTVCGVPVDLASIDVPTYIYGSREDHIVPWTA<br>AYASTALLANKLRFVLGASGHIAGVINPPAKNKRSHWTNDALPESPQQW<br>LAGAIEHHGSWWPDWTAWLAGQAGAKRAAPANYGNARYRAIEPAPGR<br>YVKAKA-Linker-<br>RVQPTESIVRFPNITNLCPFGEVFNATRFASVYAWNRKRISNCVADYSVL<br>YNSASFSTFKCYGVSPTKLNDLCFTNVYADSFVIRGDEVRQIAPGQTGKI<br>ADYNYKLPDDFTGCVIAWNSNNLDSKVGGNYNYLYRLFRKSNLKPFER<br>DISTEIYQAGSTPCNGVEGFNCYFPLQSYGFQPTNGVGYQPYRVVVLSFE<br>LLHAPATVCGPKK	PhaC: G6-R141, F146-R299, D306-F318, L329-K413, G434-W536, P549-R573, A576-R583<br><br>RBD: I624-T677, F684-R746, K750-C772, G788-K821
RBD-PhaC-N protein (135.4 kDa)	
MRVQPTESIVRFPNITNLCPFGEVFNATRFASVYAWNRKRISNCVADYSV<br>LYNSASFSTFKCYGVSPTKLNDLCFTNVYADSFVIRGDEVRQIAPGQTGKI<br>ADYNYKLPDDFTGCVIAWNSNNLDSKVGGNYNYLYRLFRKSNLKPFER<br>DISTEIYQAGSTPCNGVEGFNCYFPLQSYGFQPTNGVGYQPYRVVVLSFE<br>LLHAPATVCGPKK-Linker-<br>ATGKGAAASTQEGKSQPFKVTPGPFDPATWLEWSRQWQGTEGNGHAA<br>ASGIPGLDALAGVKIAPAQLGDIQQRYMKDFSALWQAMAEGKAEATGP<br>LHDRRFAGDAWRTNLPYRFAAAFYLLNARALTELADAVEADAKTRQRI<br>RFAISQWVDAMSPANFLATNPEAQRLLIESGGESLRAGVRNMMEDLTRG<br>KISQTDESAFEVGRNVAVTEGAVVFENEYFQLLQYKPLTDKVHARPLLM<br>VPPCINKYYILDLQPESSLVRHVVEQGHTVFLVSWRNPDASMAGSTWDD<br>YIEHAAIRAIEVARDISGQDKINVLGFCVGGTIVSTALAVLAARGEHPAAS<br>VTLLTTLLDFADTGILDVFVDEGHVQLREATLGGGAGAPCALLRGLELA<br>NTFSFLRPNDLVWNYVVDNYLKGNTPVPFDLLFWNGDATNLPGPWYC<br>WYLRHTYLQNELKVPGKLTVCGVPVDLASIDVPTYIYGSREDHIVPWTA<br>AYASTALLANKLRFVLGASGHIAGVINPPAKNKRSHWTNDALPESPQQW<br>LAGAIEHHGSWWPDWTAWLAGQAGAKRAAPANYGNARYRAIEPAPGR<br>YVKAKA-Linker-<br>SDNGPQNQRNAPRITFGGPSDSTGSNQNGERSGARSKQRRPQGLPNNTAS<br>WFTALTQHGKEDLKFPRGQGVPINTNSSPDDQIGYYRRATRRIRGGDGK<br>MKDLSPRWYFYYLGTGPEAGLPYGANKDGIIWVATEGALNTPKDHIGTR<br>NPANNAAIVLQLPQGTTLPKGFYAEGSRGGSQASSRSSSRSRNSSRNSTPG<br>SSRGTSPARMAGNGGDAALALLLLDRLNQLESKMSGKGQQQQGQTVTK<br>KSAAEASKKPRQKRTATKAYNVTQAFGRRGPEQTQGNFGDQELIRQGTD<br>YKHWPQIAQFAPSASAFFGMSRIGMEVTPSGTWLTYTGAIKLDDKDPNF<br>KDQVILLNKHIDAYKTFPPTEPKKDKKKKADETQALPQRQKKQQTVTLL<br>PAADLDDFSKQLQQSMSSADSTQA	RBD: M1-R11, I15-K39, I41-K69, A81-R137, K141-P162, G179-R192<br><br>PhaC: A218-K355, F362-R423, V451-V488, N495-R515, G552-F610, Y653-K671, L681-K714, F717-K734, R778-R788, A791-R800<br><br>N protein: I839-R913, M925-R931, Y936-K967, N974-R1001, M1034-R1117, I1144-K1194, K1198-R1209, K1212-A1243

Table S2. Genes differentially expressed on SARS-CoV-2 infection in human bronchial epithelial (NHBE) cells using GSE147507.

Gene name	log2FoldChange	p value	p_adjust	Direction
CCL20	3.14396175	3.26E-80	3.91E-76	up
SAA2	2.42248925	3.4E-79	2.04E-75	up
SAA1	2.22177266	8.61E-58	3.44E-54	up
S100A8	1.86933142	2.92E-53	6.99E-50	up
IL36G	2.73218605	2.37E-53	6.99E-50	up
TNFAIP3	1.61148647	2.41E-52	4.82E-49	up
SPRR2D	2.97919497	1.76E-51	3.01E-48	up
INHBA	1.81922735	5.18E-51	7.76E-48	up
C3	1.50866591	4.32E-46	5.76E-43	up
KRT6B	1.54827214	9.85E-46	1.18E-42	up
ICAM1	1.86166523	1.76E-45	1.76E-42	up
TNIP1	1.26402353	1.71E-45	1.76E-42	up
CFB	1.8548093	2.18E-44	2.01E-41	up
CXCL1	1.4172731	2.58E-42	2.21E-39	up
SOD2	1.51945874	3.03E-38	2.42E-35	up
MX1	2.50985094	1.38E-36	1.04E-33	up
S100A9	1.10738825	5.9E-36	4.16E-33	up

ZC3H12A	1.67390106	1.13E-34	7.51E-32	up
C15orf48	1.23597139	2.1E-31	1.32E-28	up
LIF	1.30739366	4.38E-31	2.63E-28	up
CXCL5	3.48535834	7.82E-31	4.47E-28	up
RHCG	1.34872189	8.28E-31	4.52E-28	up
HBEGF	1.29188055	1.31E-30	6.84E-28	up
SLC6A14	1.21811219	2.83E-29	1.42E-26	up
IRF9	1.25047085	9.16E-29	4.39E-26	up
IL1B	1.06251861	4.02E-28	1.85E-25	up
SPRR2A	1.68768801	3.1E-27	1.38E-24	up
TNFAIP2	1.54932822	1.73E-26	7.39E-24	up
IL6	2.92646346	2.12E-26	8.77E-24	up
MMP9	2.28503359	1.42E-25	5.66E-23	up
IL32	1.22070157	1.7E-24	6.6E-22	up
SERPINA3	1.46770413	9.92E-24	3.72E-21	up
PDZK1IP1	2.4278506	2.79E-23	1.02E-20	up
OAS3	1.31779675	2.92E-23	1.03E-20	up
PLAT	1.39509077	1.09E-22	3.75E-20	up
CSF3	4.85032851	1.15E-22	3.82E-20	up
BIRC3	1.66152604	1.74E-22	5.64E-20	up
OAS2	1.2662775	2.84E-22	8.98E-20	up
OAS1	1.6954135	2.56E-19	7.68E-17	up
CXCL2	1.39127199	5.35E-19	1.57E-16	up
IRAK2	1.59984891	1.5E-18	4.19E-16	up
PGLYRP4	2.03747854	6.51E-18	1.74E-15	up
CXCL3	2.23021571	2.93E-17	7.64E-15	up
MAFF	1.13326426	6.1E-17	1.56E-14	up
SPRR2E	3.59793307	1.92E-16	4.8E-14	up
IL1A	1.07203604	7.41E-16	1.74E-13	up
HEPHL1	1.30788314	1.43E-15	3.29E-13	up
XAF1	2.53063831	1.98E-15	4.48E-13	up
IVL	1.03806572	5.92E-15	1.29E-12	up
IFI27	2.89474326	1.2E-14	2.58E-12	up
C1QTNF1	1.87212235	1.52E-14	3.19E-12	up
CSF2	2.97894511	5.15E-13	1.05E-10	up
IFITM10	-1.5710814	1.63E-12	3.27E-10	down
BCL2A1	2.28834639	2.52E-12	4.95E-10	up
IFITM1	1.6147879	1.18E-11	2.14E-09	up
EDN1	1.0605424	3.5E-10	5.83E-08	up
PI3	1.80075642	1.75E-09	2.56E-07	up
PDGFB	1.01434311	1.8E-09	2.61E-07	up
MX2	2.21993955	2.83E-09	3.99E-07	up
SOCS3	1.0373848	2.35E-08	0.00000285	up
VNN1	1.883174	3.53E-08	0.00000412	up
KRT4	1.01298875	4.22E-08	0.00000482	up
TYMP	1.04040175	4.89E-08	0.00000553	up
VTCN1	-1.0742581	6.03E-08	0.00000676	down
MYLK	-1.4288626	6.57E-08	0.00000714	down
CXCL14	-1.4485315	6.6E-08	0.00000714	down
METTL7A	-1.0357868	1.44E-07	0.0000149	down
ADAM8	1.01426869	1.94E-07	0.0000192	up
ITGB3	1.19799377	9.47E-07	0.0000836	up
CYP27B1	1.20606261	0.00000133	0.000113	up
TNFSF14	1.99248464	0.0000014	0.00011664	up
RND1	1.51644941	0.00000195	0.00015935	up
IRF7	1.57303744	0.00000278	0.00021648	up
TNF	1.80716236	0.00000394	0.00029508	up

MYEOV	1.28223564	0.00000483	0.0003513	up
KRT24	1.79683578	0.00000869	0.00059256	up
MAP7D2	-1.3954685	0.00000867	0.00059256	down
NID1	-1.2290109	0.00000976	0.00065773	down
HELZ2	1.01051942	0.0000232	0.00142745	up
VNN3	2.74162665	0.000026	0.00157425	up
MRGPRX3	1.95888146	0.000027	0.00162927	up
IFI6	2.34449344	0.0000344	0.00202474	up
PPARGC1A	-1.0471203	0.0000363	0.00210458	down
TRIML2	1.39749281	0.0000463	0.00257094	up
TLR2	1.05056807	0.0000486	0.00268432	up
BST2	1.29187352	0.0000521	0.0028396	up
MAP3K8	1.06163063	0.0000574	0.00308576	up
EPSTI1	1.04447947	0.0000608	0.00324312	up
ATG9B	-1.1310397	0.0000614	0.0032589	down
GBP5	1.83077415	0.00012273	0.00603509	up
CXCL6	1.73041085	0.00012274	0.00603509	up
NANOS1	-1.5176398	0.00012404	0.00607391	down
S100A12	1.2238146	0.00017339	0.00809391	up
LTB	1.6385608	0.00018339	0.00849455	up
STAT5A	1.11992847	0.00018495	0.00853402	up
STON1	-1.5080852	0.00019045	0.00872086	down
IFI44L	2.27820079	0.00028877	0.01246185	up
MMP13	1.09446902	0.00030101	0.01285144	up
ZNF488	-1.1399958	0.00037534	0.01516131	down
CYP4F3	-1.0730949	0.00041372	0.01632709	down
FAM167A	1.09674174	0.00082419	0.02843076	up
P2RY6	1.13520504	0.00100864	0.03279294	up
RBM20	-1.5835055	0.00127333	0.03967835	down
PSMB9	1.01490868	0.00151653	0.04497961	up
C6orf223	1.15507754	0.00156657	0.04601422	up

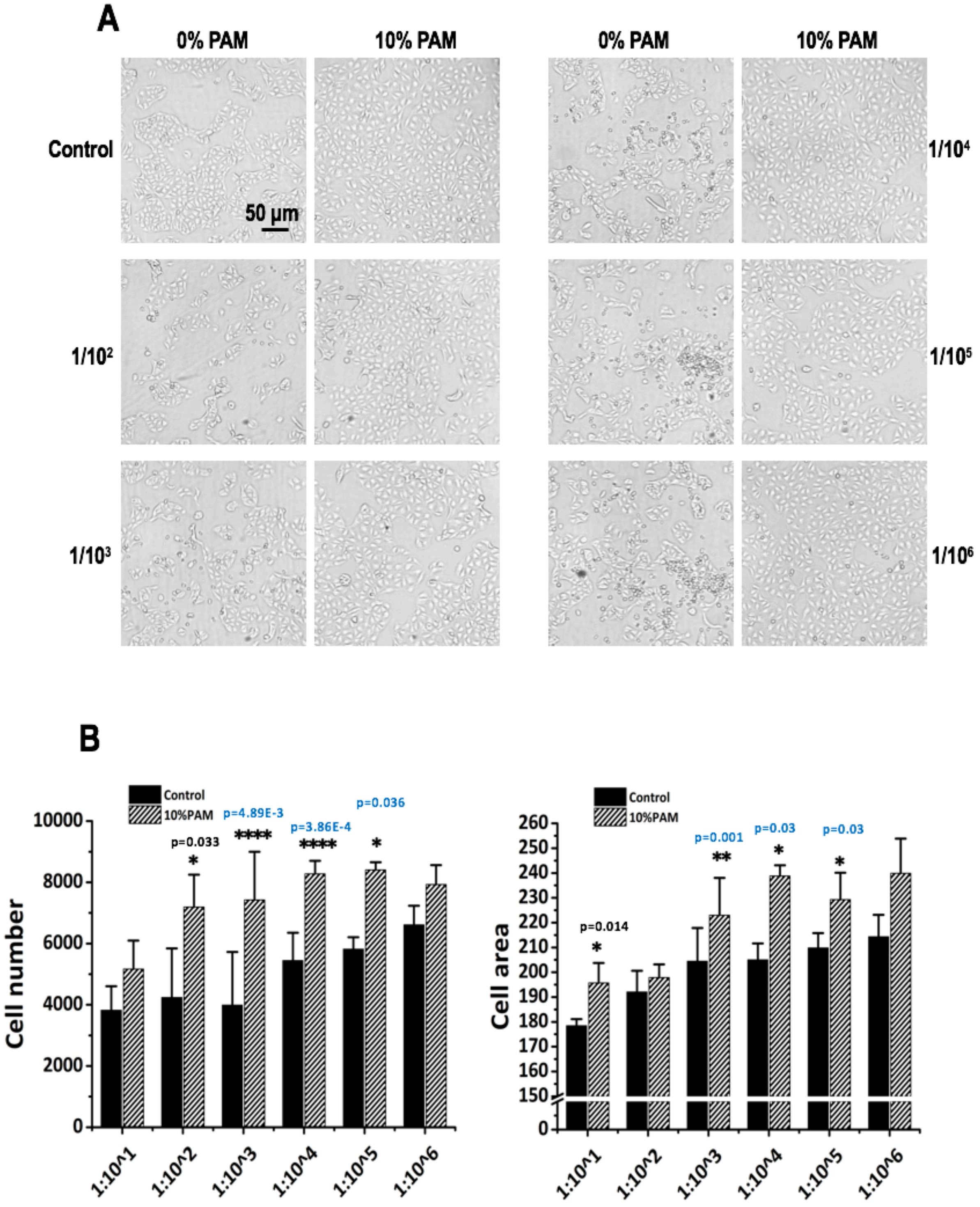

Figure S1. Images showing the preventive role of CAP on Vero E6 cells against SARS-CoV-2 infection. (A) Phase contrast images of cells infected with different dilution of 10E6.79 TCID50/mL virus, without (0% PAM) or with (10% PAM) pre-treatment, prior to virus addition. Cell morphology alterations were observed 1 day after exposing cells to 10 min 10% PAM treatment. (B) Graphs show quantification of cell number and cell area, conducted using IN Carta Analysis, with statistical analysis (n=4) using t-test assuming unequal variance or non-parametric tests, as appropriate. P values are shown above the bars in red if significant. Images of cells infected with SARS-CoV-2 at different dilution folds, without (Control) and with 10% PAM treatment prior to virus addition (10% PAM). Cell morphology alterations were observed 1 day after exposing cells to 10 min 10% PAM treatment. Quantification of cell number and cell area were conducted using IN Carta Analysis.

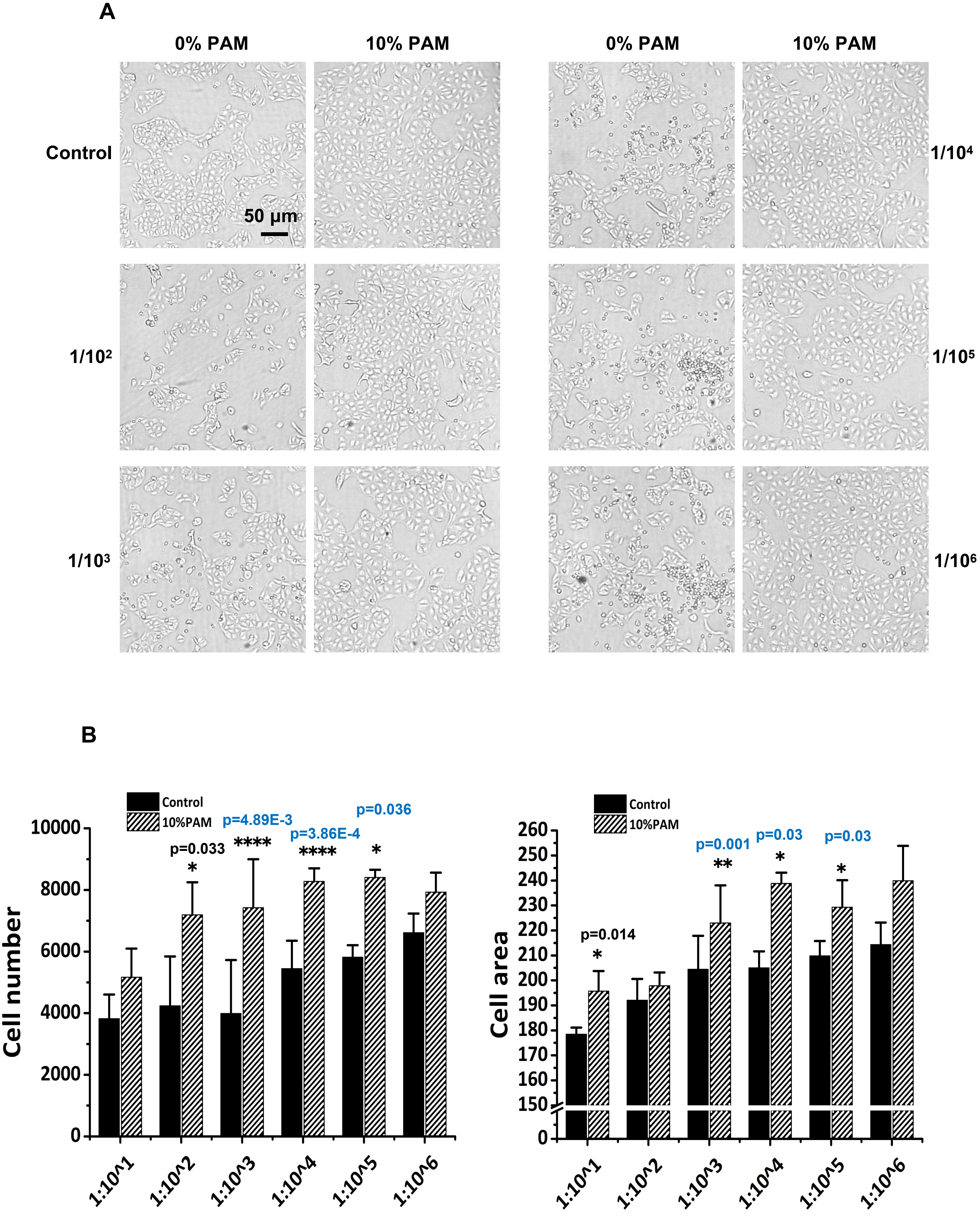
A
0% PAM
10% PAM
0% PAM
10% PAM
Control
50 µm
1/10²
1/10³
1/10⁴
1/10⁵
1/10⁶
B
Control
10%PAM
Cell number
p=0.033
p=4.89E-3
p=3.86E-4
p=0.036
1:10^1
1:10^2
1:10^3
1:10^4
1:10^5
1:10^6
Cell area
p=0.014
p=0.001
p=0.03
p=0.03

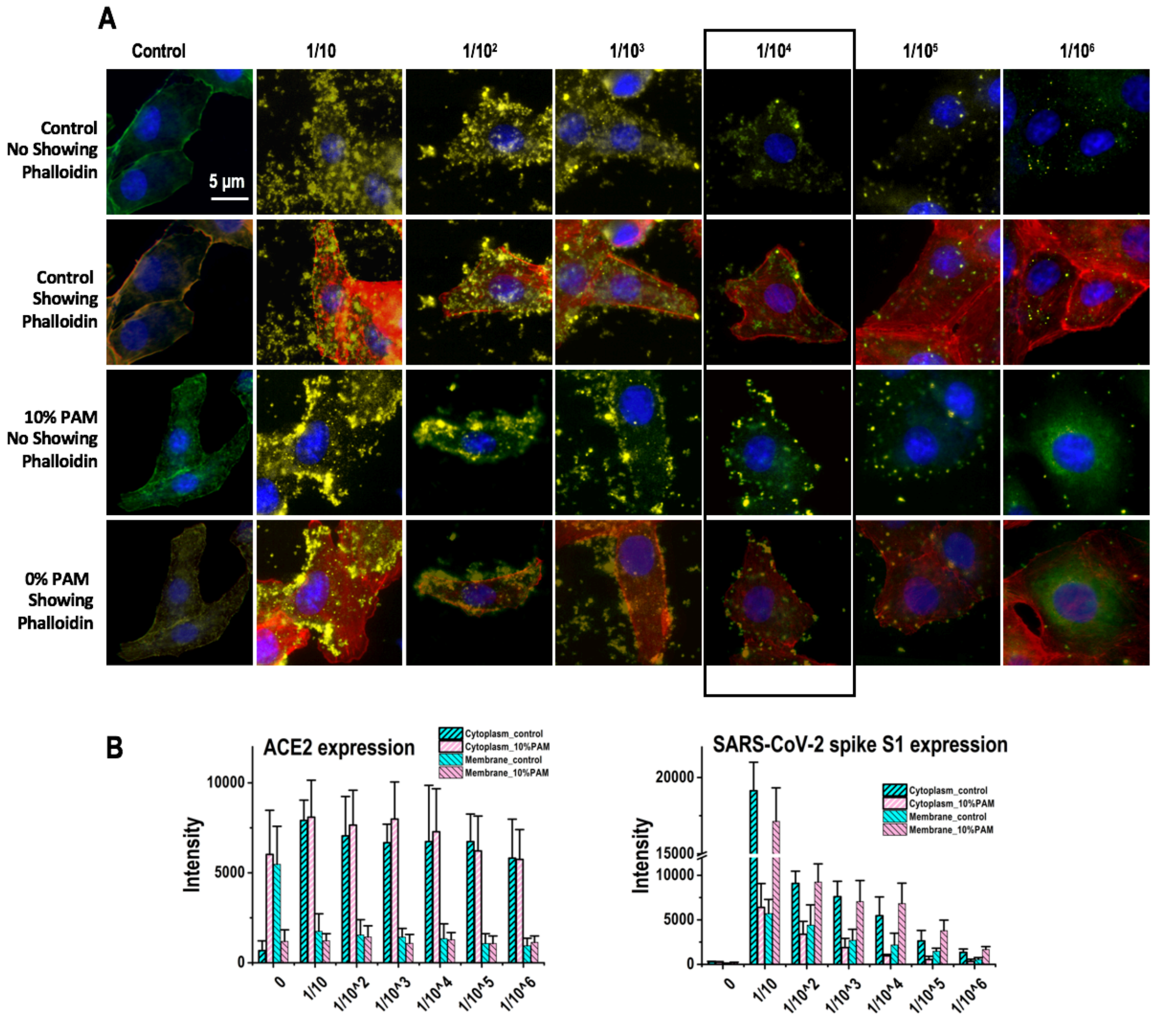

p (Cytoplasm vs. Membrane)

	0	$1/10^1$	$1/10^2$	$1/10^3$	$1/10^4$	$1/10^5$	$1/10^6$
Control	1.4E-3	1.2E-4	1.1E-3	8.0E-5	3.7E-3	2.3E-4	1.3E-3
PAM	2.3E-3	3.0E-4	3.9E-4	3.1E-4	8.7E-4	7.2E-4	6.1E-4
	*	*	*	*	*	*	*

p (PAM vs. Control)

	0	$1/10^1$	$1/10^2$	$1/10^3$	$1/10^4$	$1/10^5$	$1/10^6$
Cytoplasm	0.276	1.5E-4	5.1E-4	5.3E-4	1.5E-3	3.5E-3	1.3E-3
Membrane	0.087	1.1E-4	4.5E-3	4.1E-3	3.1E-3	2.4E-3	4.2E-4
		*	*	*	*	*	*

Figure S2. Additional images showing the preventive role of CAP on Vero E6 cells against SARS-CoV-2 infection. (A) Immunofluorescence for ACE2 (green) or SARS-CoV-2 viral particles (yellow) and staining for actin filaments (phalloidin; red) or DNA (Hoechst 33342; blue), showing extracellular virus on Vero E6 cells that underwent 0% PAM or 10% PAM pre-treatment prior to the infection with different dilution of 10E6.79 TCID50/mL SARS-CoV-2 virus. (B) Phalloidin staining of actin was used as a reference against which the number and intensity of stained particles were counted. Graphs show quantification of ACE2 staining or SARS-CoV-2 virus particles, conducted using IN Carta Analysis, and represent mean -/+ SD of the data. Significance was assessed using Student's T-test and p-values for each comparison (0% vs 10% PAM) are shown in the Tables below each graph. n=4.

A

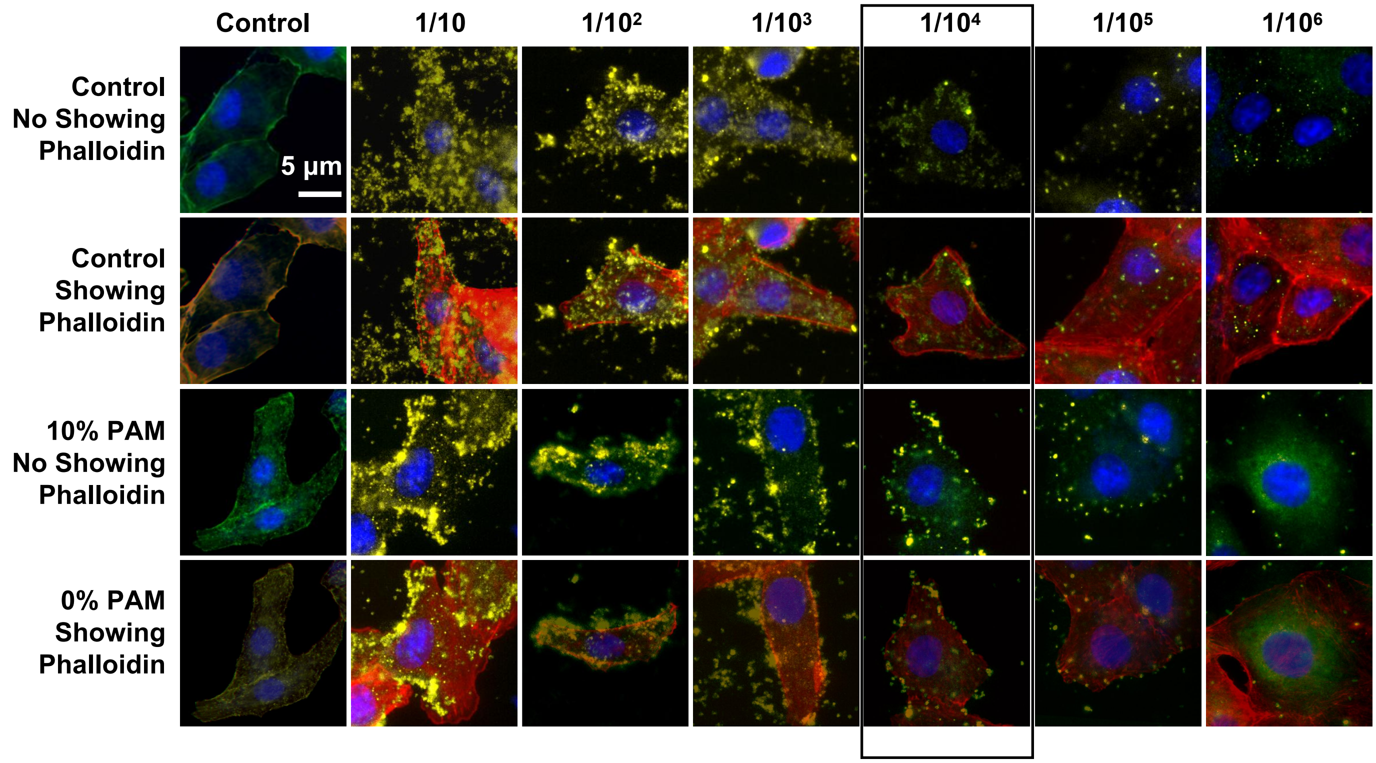

B

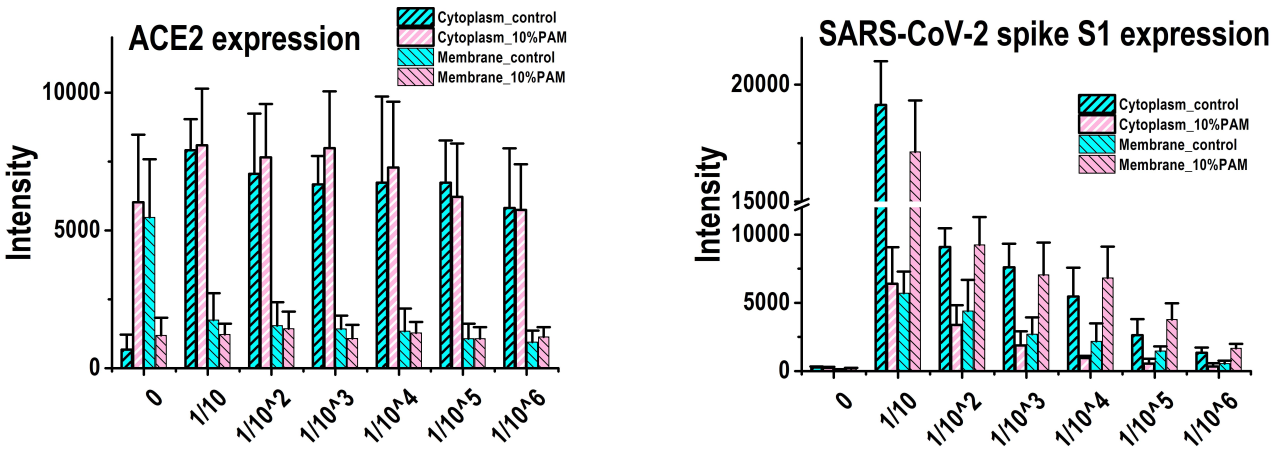

p (Cytoplasm vs. Membrane)

	0	$1/10^1$	$1/10^2$	$1/10^3$	$1/10^4$	$1/10^5$	$1/10^6$
Control	1.4E-3	1.2E-4	1.1E-3	8.0E-5	3.7E-3	2.3E-4	1.3E-3
PAM	2.3E-3	3.0E-4	3.9E-4	3.1E-4	8.7E-4	7.2E-4	6.1E-4
	*	*	*	*	*	*	*

p (PAM vs. Control)

	0	$1/10^1$	$1/10^2$	$1/10^3$	$1/10^4$	$1/10^5$	$1/10^6$
Cytoplasm	0.276	1.5E-4	5.1E-4	5.3E-4	1.5E-3	3.5E-3	1.3E-3
Membrane	0.087	1.1E-4	4.5E-3	4.1E-3	3.1E-3	2.4E-3	4.2E-4
		*	*	*	*	*	*

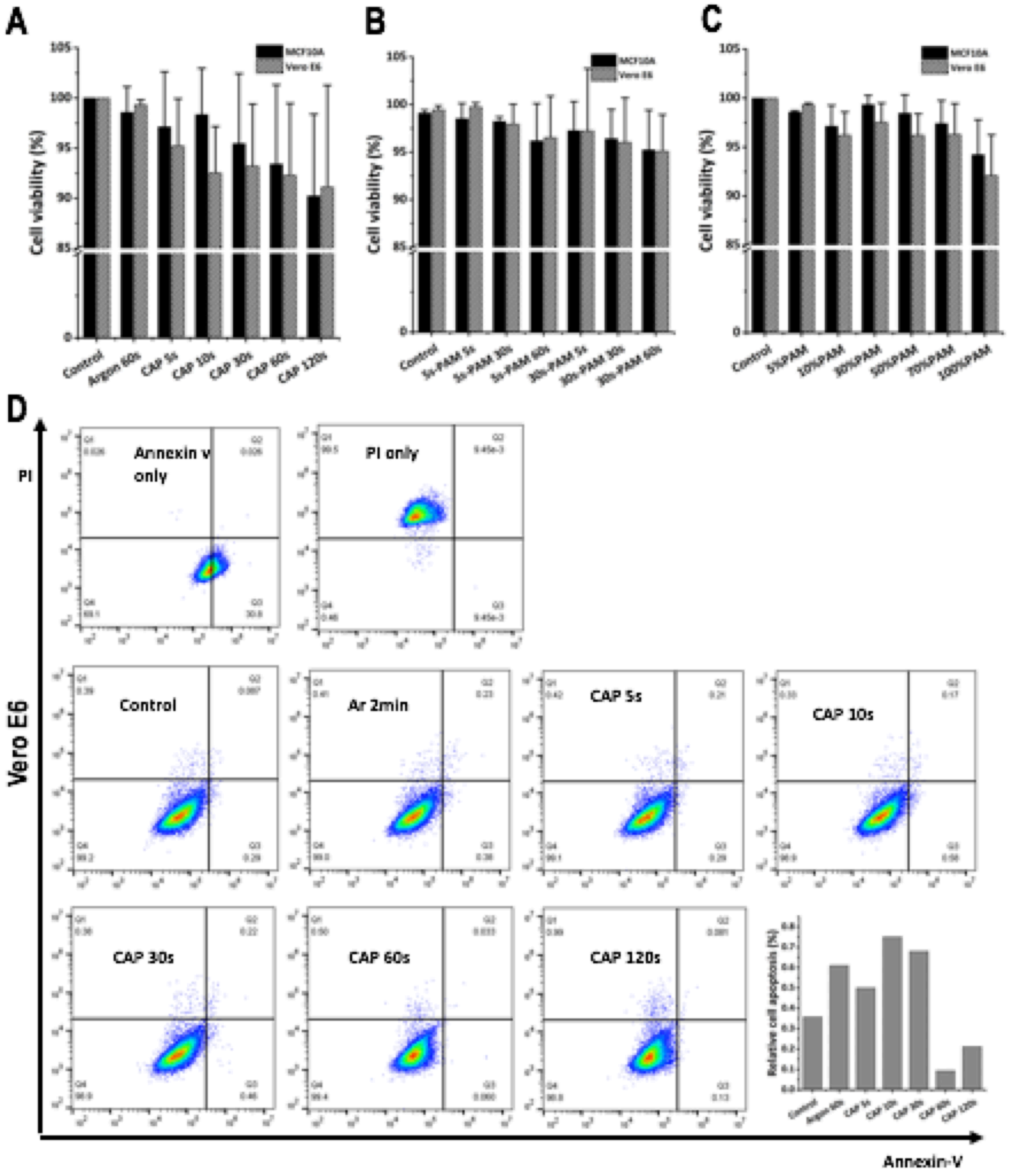
A
B
C
D
MCF10A
Vero E6
Cell viability (%)
Control
Argon 60s
CAP 5s
CAP 10s
CAP 30s
CAP 60s
CAP 120s
5s-PAM 5s
5s-PAM 30s
5s-PAM 60s
30s-PAM 5s
30s-PAM 30s
30s-PAM 60s
5%PAM
10%PAM
30%PAM
50%PAM
70%PAM
100%PAM
PI
Annexin v only
PI only
Vero E6
Control
Ar 2min
CAP 5s
CAP 10s
CAP 30s
CAP 60s
CAP 120s
Relative cell apoptosis (%)
Annexin-V

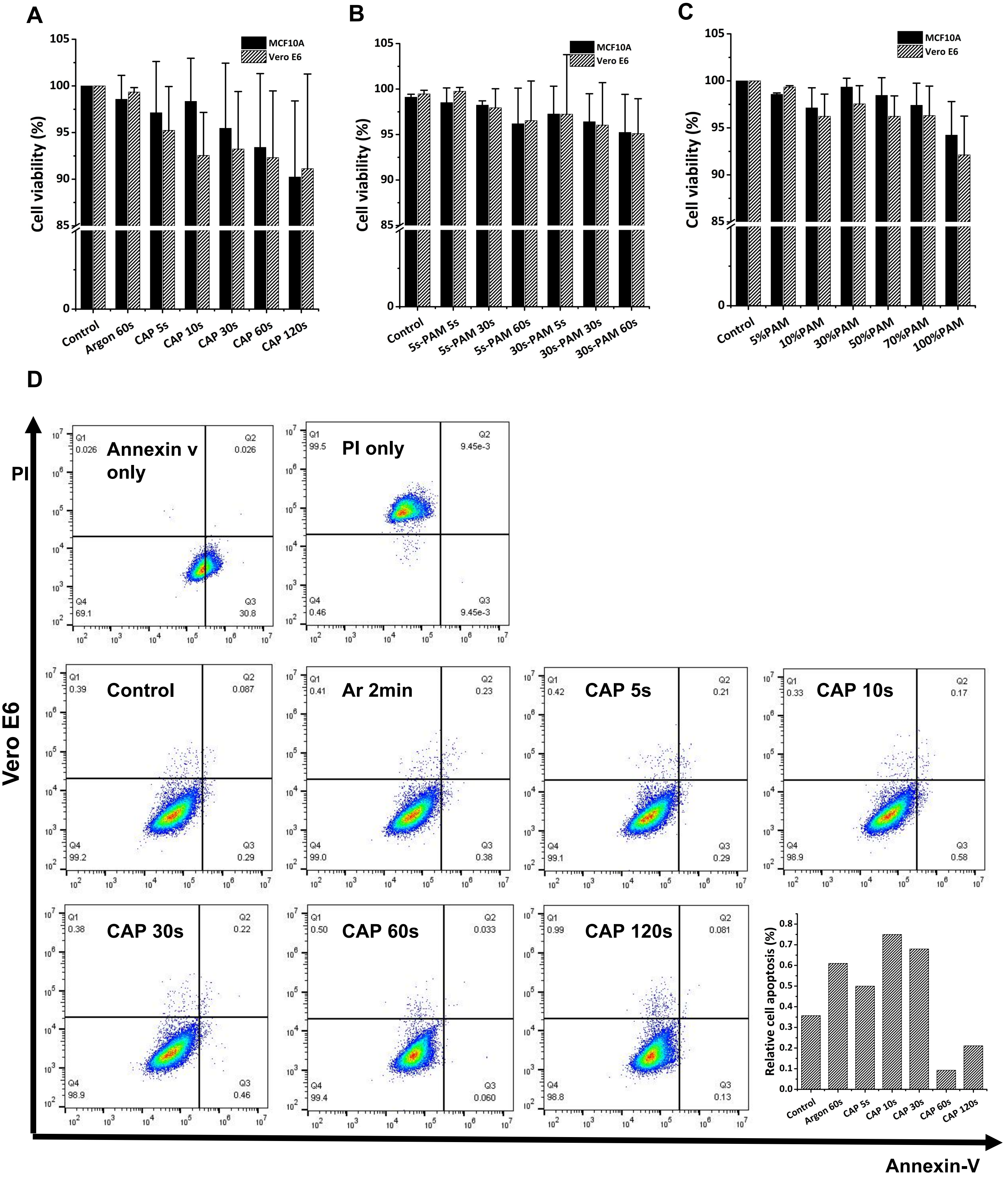
A
B
C
D
MCF10A
Vero E6
Cell viability (%)
105
100
95
90
85
0
Control
Argon 60s
CAP 5s
CAP 10s
CAP 30s
CAP 60s
CAP 120s
5s-PAM 5s
5s-PAM 30s
5s-PAM 60s
30s-PAM 5s
30s-PAM 30s
30s-PAM 60s
5%PAM
10%PAM
30%PAM
50%PAM
70%PAM
100%PAM
PI
Vero E6
Annexin-V
Annexin v only
Q1 0.026
Q2 0.026
Q4 69.1
Q3 30.8
PI only
Q1 99.5
Q2 9.45e-3
Q4 0.46
Q3 9.45e-3
Control
Q1 0.39
Q2 0.087
Q4 99.2
Q3 0.29
Ar 2min
Q1 0.41
Q2 0.23
Q4 99.0
Q3 0.38
CAP 5s
Q1 0.42
Q2 0.21
Q4 99.1
Q3 0.29
CAP 10s
Q1 0.33
Q2 0.17
Q4 98.9
Q3 0.58
CAP 30s
Q1 0.38
Q2 0.22
Q4 98.9
Q3 0.46
CAP 60s
Q1 0.50
Q2 0.033
Q4 99.4
Q3 0.060
CAP 120s
Q1 0.99
Q2 0.081
Q4 98.8
Q3 0.13
Relative cell apoptosis (%)
0.0
0.1
0.2
0.3
0.4
0.5
0.6
0.7
0.8
Control
Argon 60s
CAP 5s
CAP 10s
CAP 30s
CAP 60s
CAP 120s

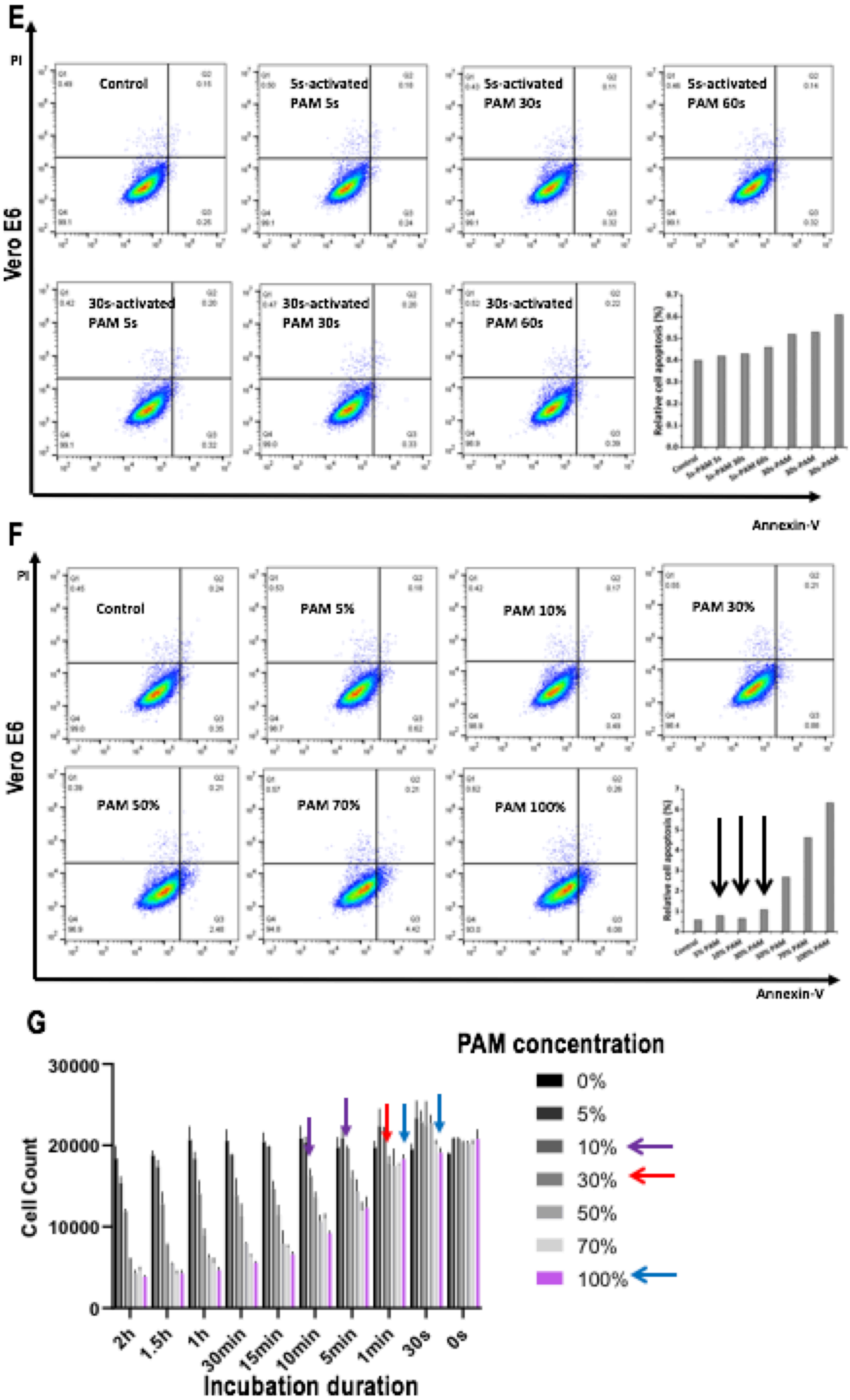
E
PI
Vero E6
Control
5s-activated PAM 5s
5s-activated PAM 30s
5s-activated PAM 60s
30s-activated PAM 5s
30s-activated PAM 30s
30s-activated PAM 60s
Annexin-V
F
PI
Vero E6
Control
PAM 5%
PAM 10%
PAM 30%
PAM 50%
PAM 70%
PAM 100%
Annexin-V
G
PAM concentration
0%
5%
10%
30%
50%
70%
100%
Cell Count
30000
20000
10000
0
2h
1.5h
1h
30min
15min
10min
5min
1min
30s
0s
Incubation duration

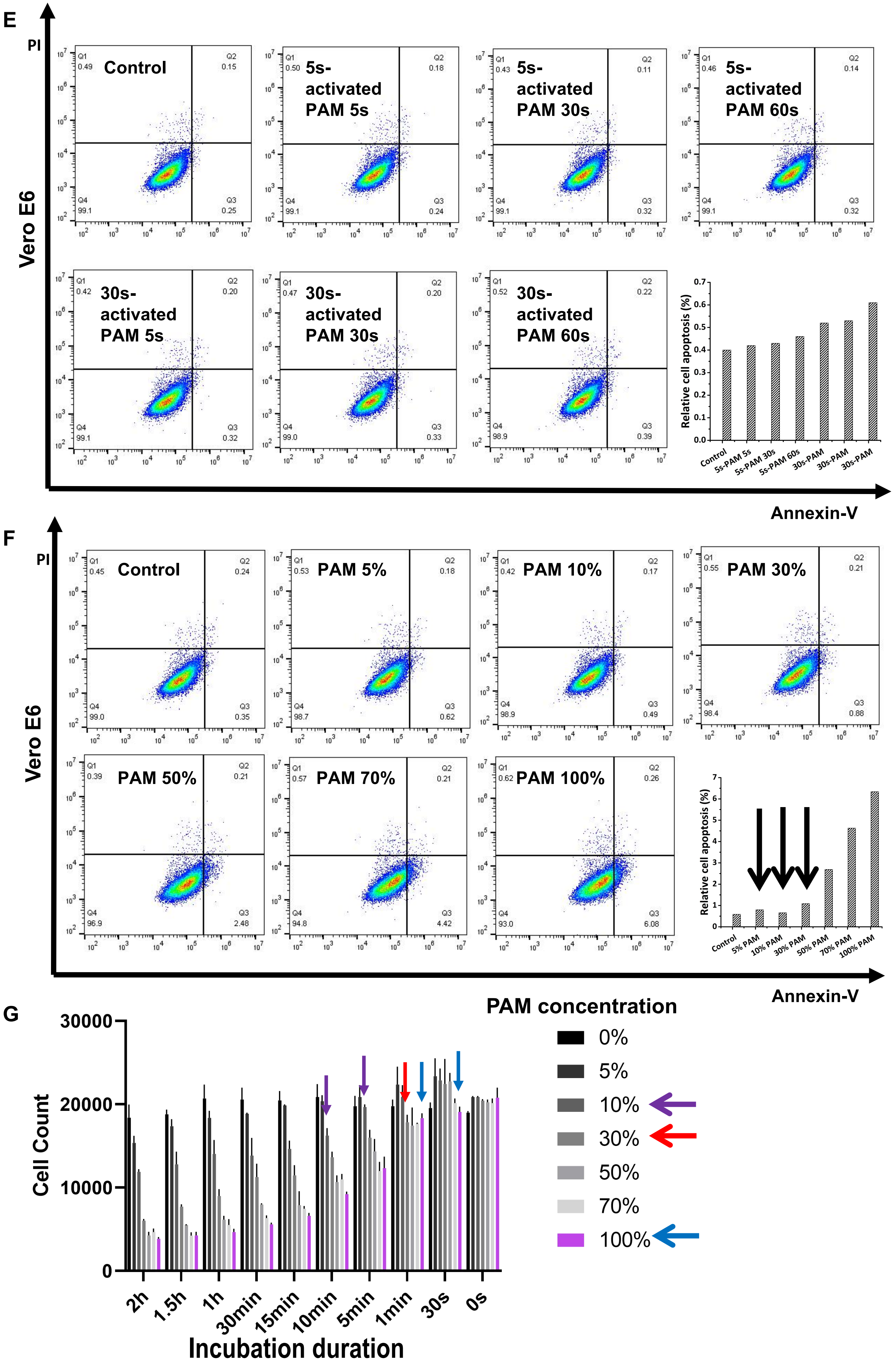
E
PI
Vero E6
Control
5s-activated PAM 5s
5s-activated PAM 30s
5s-activated PAM 60s
30s-activated PAM 5s
30s-activated PAM 30s
30s-activated PAM 60s
Relative cell apoptosis (%)
Control
5s-PAM 5s
5s-PAM 30s
5s-PAM 60s
30s-PAM
30s-PAM
30s-PAM
Annexin-V
F
PI
Vero E6
Control
PAM 5%
PAM 10%
PAM 30%
PAM 50%
PAM 70%
PAM 100%
Relative cell apoptosis (%)
Control
5% PAM
10% PAM
30% PAM
50% PAM
70% PAM
100% PAM
Annexin-V
G
Cell Count
PAM concentration
0%
5%
10%
30%
50%
70%
100%
2h
1.5h
1h
30min
15min
10min
5min
1min
30s
0s
Incubation duration

Figure S3. Additional images showing the effects of different CAP and PAM concentrations on cell viability and apoptosis. Viability of MCF10A and Vero E6 cells in response to (A) direct CAP treatment at different treatment durations, (B) indirect PAM treatment activated by CAP for different treatment durations, and (C) indirect 10-min activated PAM treatment diluted into different concentrations. Apoptosis of Vero E6 cells in response to (D) different CAP treatment durations, (E) different CAP exposure time activated PAM for different treatment durations, and (F) 1 min CAP exposure activated PAM diluted into different concentrations. (G) Vero E6 cell counts under PAM indirect treatment at different diluted concentrations and for different treatment durations.

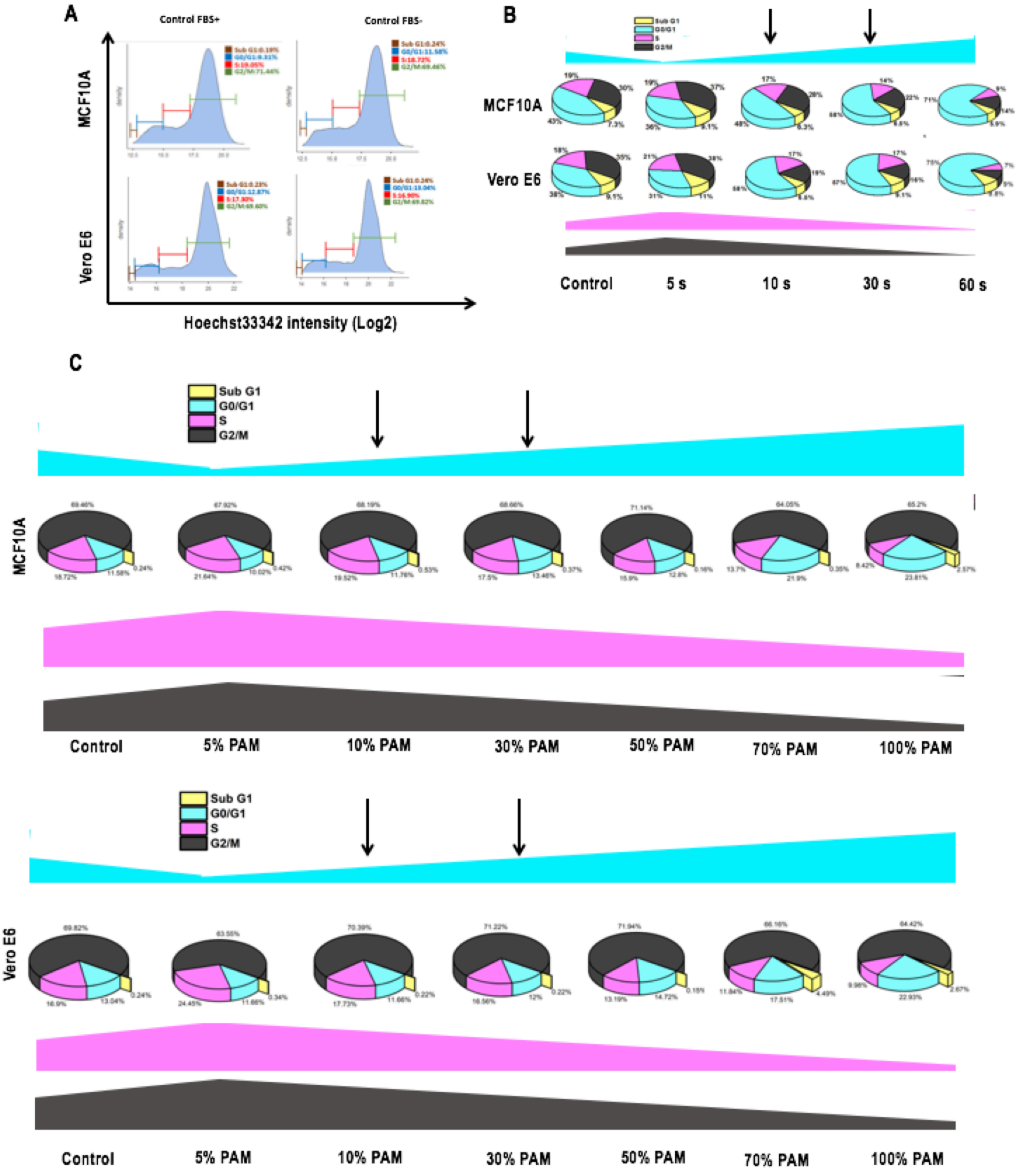

Figure S4. Additional images showing the effects of different CAP and PAM concentrations on cell cycle. (A) Effect of FCS on cell cycle in MCF10A and Vero E6 cells. Cell cycle alteration in response to (B) direct CAP treatment at different treatment durations in MCF10A and Vero E6 cells, and (C) indirect 10-min activated PAM treatment diluted into different concentrations in MCF10A and Vero E6 cells.

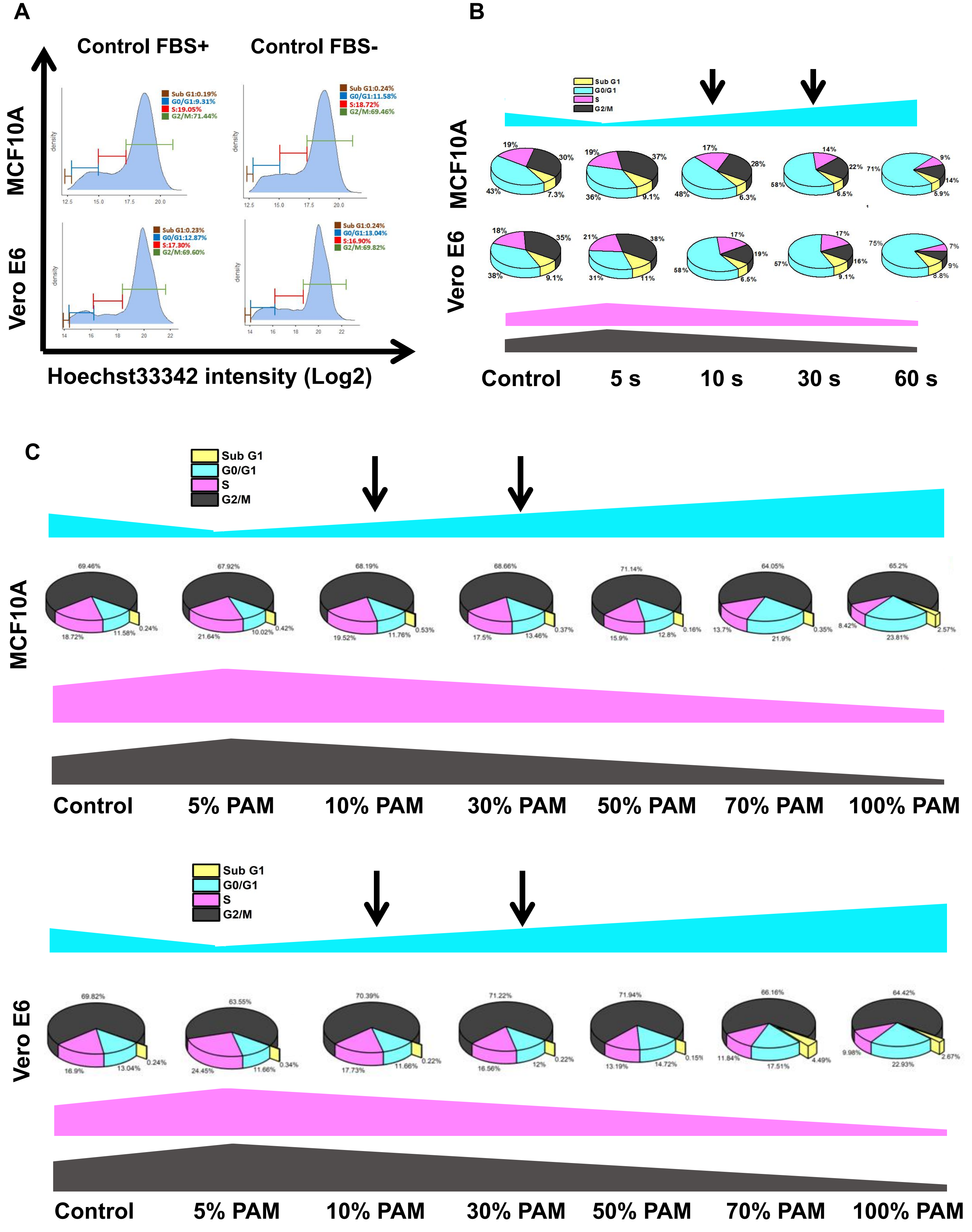

A
Control FBS+
Control FBS-
MCF10A
Vero E6
Sub G1:0.19%
G0/G1:9.31%
S:19.05%
G2/M:71.44%
Sub G1:0.24%
G0/G1:11.58%
S:18.72%
G2/M:69.46%
Sub G1:0.23%
G0/G1:12.87%
S:17.30%
G2/M:69.60%
Sub G1:0.24%
G0/G1:13.04%
S:16.90%
G2/M:69.82%
density
Hoechst33342 intensity (Log2)
B
Sub G1
G0/G1
S
G2/M
MCF10A
Vero E6
Control
5 s
10 s
30 s
60 s
C
Sub G1
G0/G1
S
G2/M
MCF10A
Control
5% PAM
10% PAM
30% PAM
50% PAM
70% PAM
100% PAM
Sub G1
G0/G1
S
G2/M
Vero E6
Control
5% PAM
10% PAM
30% PAM
50% PAM
70% PAM
100% PAM

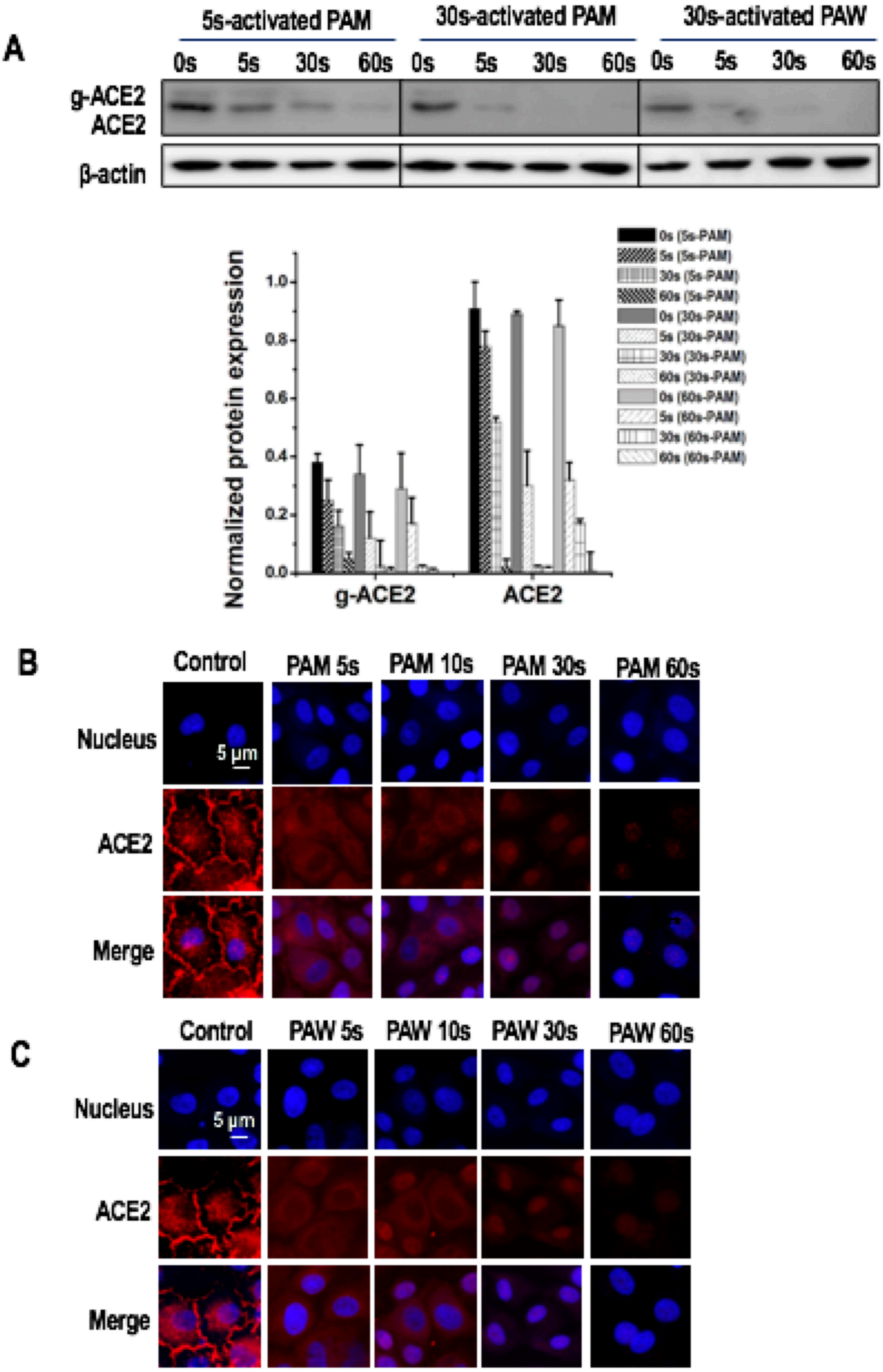

Figure S5. Additional immunofluorescence and Western blots images showing ACE2 expression and cell localization after indirect CAP exposure for different durations.

(A) Western blot of indirect PAM/PAW treatment.

(B) Quantification of ACE2 on indirect PAM/PAW treatment.

(C) Immunofluorescence images of ACE2 on indirect PAM treatment.

(D) Immunofluorescence images of ACE2 on indirect PAW treatment. PAM = plasma activated medium; PAW = plasma activated PBS. MCF10A cells were used. Scale bar=5 µm.

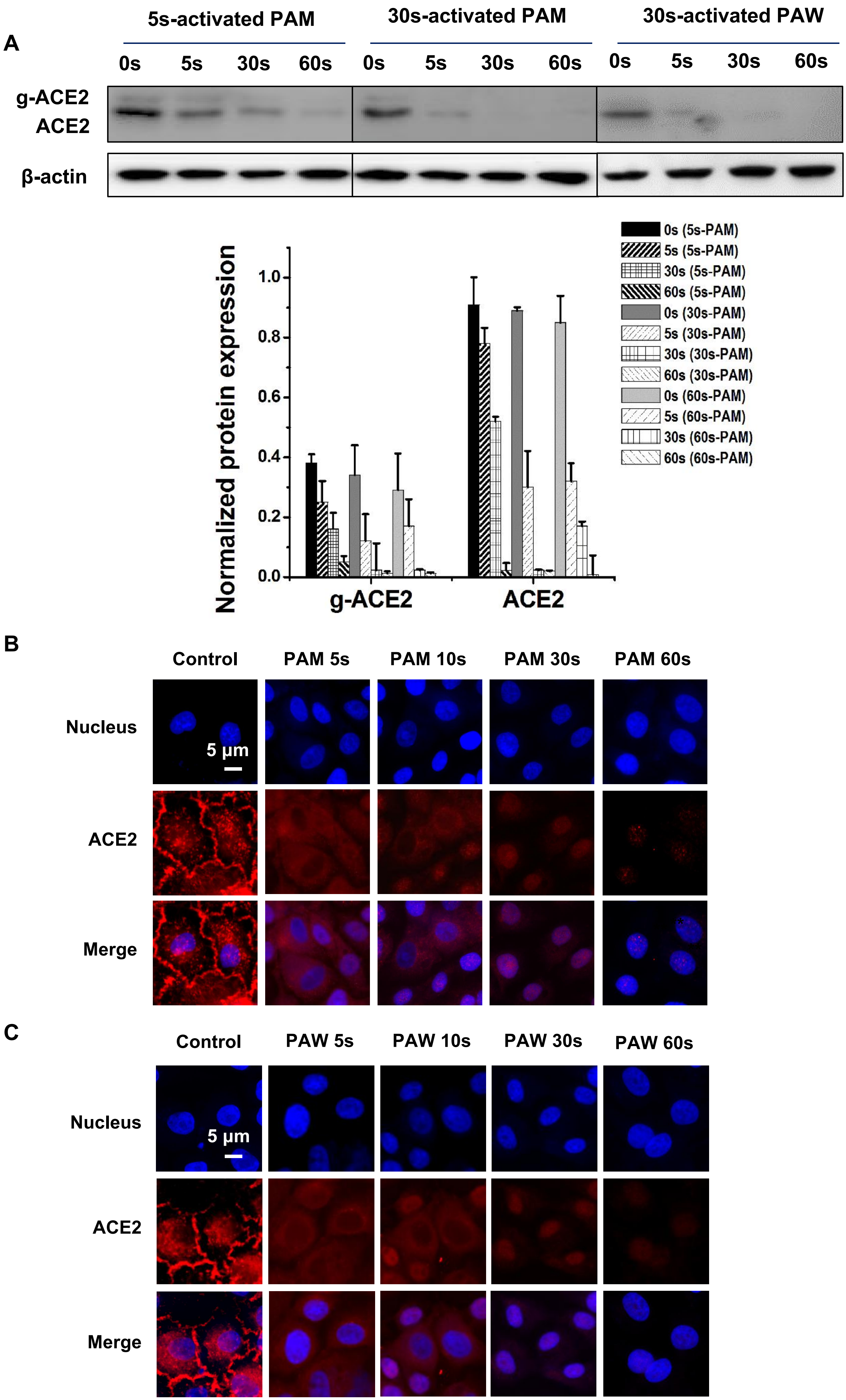

A
5s-activated PAM
30s-activated PAM
30s-activated PAW
0s
5s
30s
60s
g-ACE2
ACE2
β-actin
Normalized protein expression
1.0
0.8
0.6
0.4
0.2
0.0
g-ACE2
ACE2
0s (5s-PAM)
5s (5s-PAM)
30s (5s-PAM)
60s (5s-PAM)
0s (30s-PAM)
5s (30s-PAM)
30s (30s-PAM)
60s (30s-PAM)
0s (60s-PAM)
5s (60s-PAM)
30s (60s-PAM)
60s (60s-PAM)
B
Control
PAM 5s
PAM 10s
PAM 30s
PAM 60s
Nucleus
5 μm
ACE2
Merge
C
Control
PAW 5s
PAW 10s
PAW 30s
PAW 60s
Nucleus
5 μm
ACE2
Merge

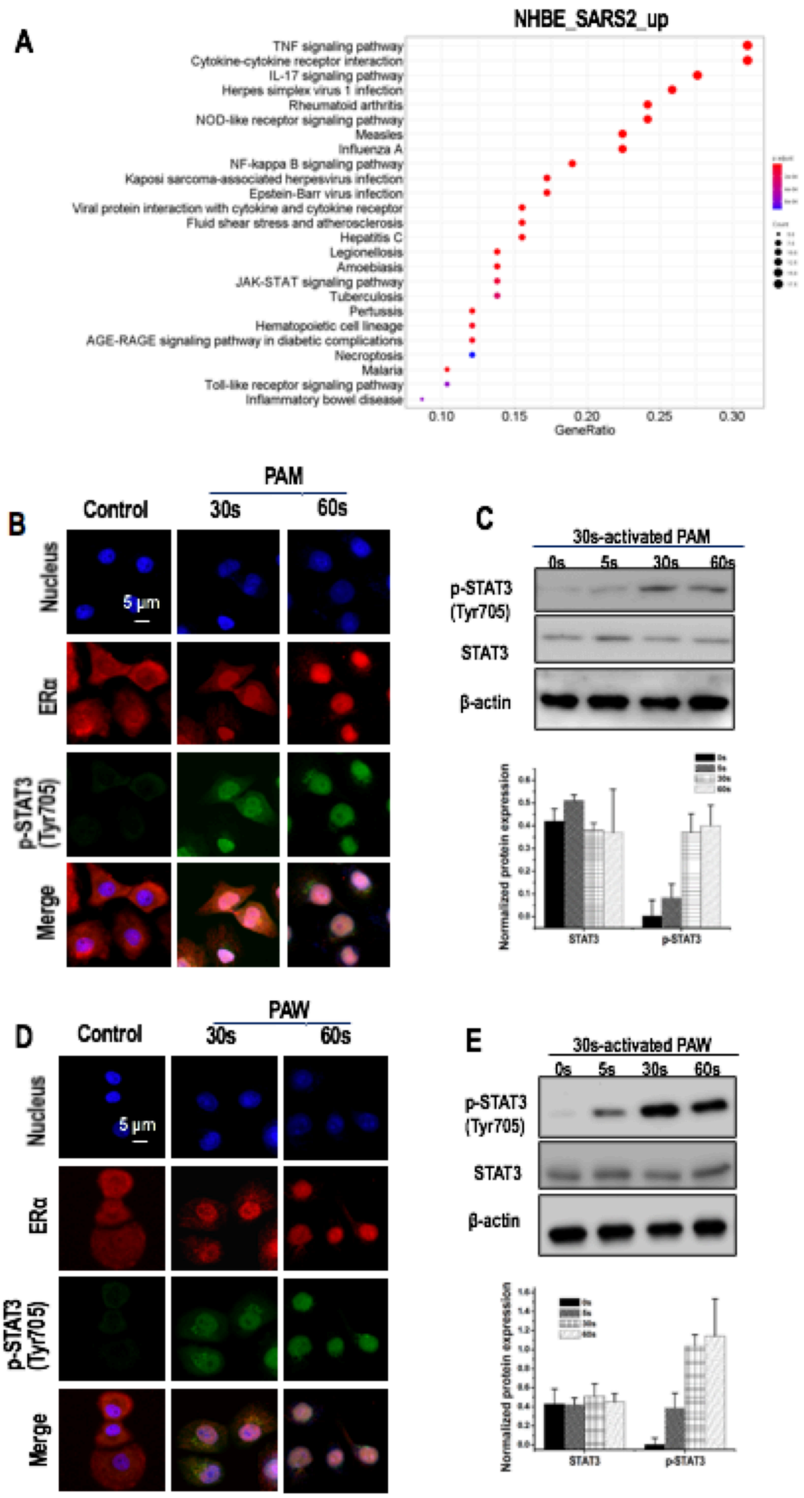

Figure S6. Additional immunofluorescence and western blot images showing ERα and STAT3(Tyr705) expression and cell localization after indirect CAP exposure under different durations.
(A) Up-regulated KEGG pathways enriched by differentially expressed genes in human bronchial epithelial (NHBE) cells on SARS-CoV-2 infection.
(B) Immunofluorescence images showing ERα, phospho-STAT3(Tyr705) location after indirect PAM treatment for different durations.
(C) Western blot and quantification showing phospho-STAT3(Tyr705) expression after indirect PAM treatment for different durations.
(D) Immunofluorescence image showing ERα, phospho-STAT3(Tyr705) location after indirect PAW treatment for different durations.
(E) Western blot and quantification showing phospho-STAT3(Tyr705) expression after indirect PAW treatment for different durations. PAM = plasma activated medium; PAW = plasma activated PBS. MCF10A cells were used.

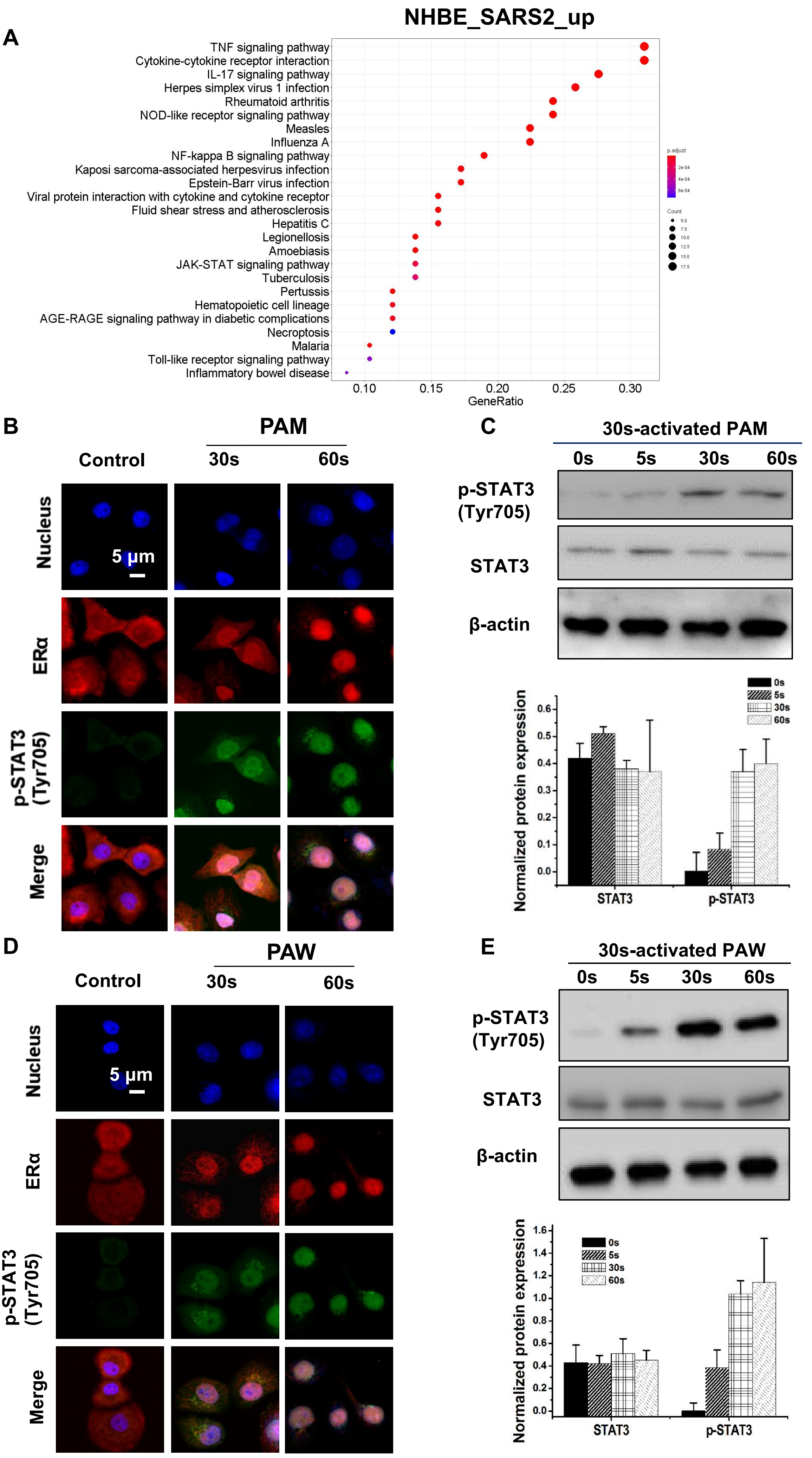
A
NHBE_SARS2_up
TNF signaling pathway
Cytokine-cytokine receptor interaction
IL-17 signaling pathway
Herpes simplex virus 1 infection
Rheumatoid arthritis
NOD-like receptor signaling pathway
Measles
Influenza A
NF-kappa B signaling pathway
Kaposi sarcoma-associated herpesvirus infection
Epstein-Barr virus infection
Viral protein interaction with cytokine and cytokine receptor
Fluid shear stress and atherosclerosis
Hepatitis C
Legionellosis
Amoebiasis
JAK-STAT signaling pathway
Tuberculosis
Pertussis
Hematopoietic cell lineage
AGE-RAGE signaling pathway in diabetic complications
Necroptosis
Malaria
Toll-like receptor signaling pathway
Inflammatory bowel disease
0.10
0.15
0.20
0.25
0.30
GeneRatio
p.adjust
Count
B
PAM
Control
30s
60s
Nucleus
5 μm
ERα
p-STAT3 (Tyr705)
Merge
C
30s-activated PAM
0s
5s
30s
60s
p-STAT3 (Tyr705)
STAT3
β-actin
Normalized protein expression
0.0
0.1
0.2
0.3
0.4
0.5
0.6
STAT3
p-STAT3
D
PAW
Control
30s
60s
Nucleus
5 μm
ERα
p-STAT3 (Tyr705)
Merge
E
30s-activated PAW
0s
5s
30s
60s
p-STAT3 (Tyr705)
STAT3
β-actin
Normalized protein expression
0.0
0.2
0.4
0.6
0.8
1.0
1.2
1.4
1.6
STAT3
p-STAT3

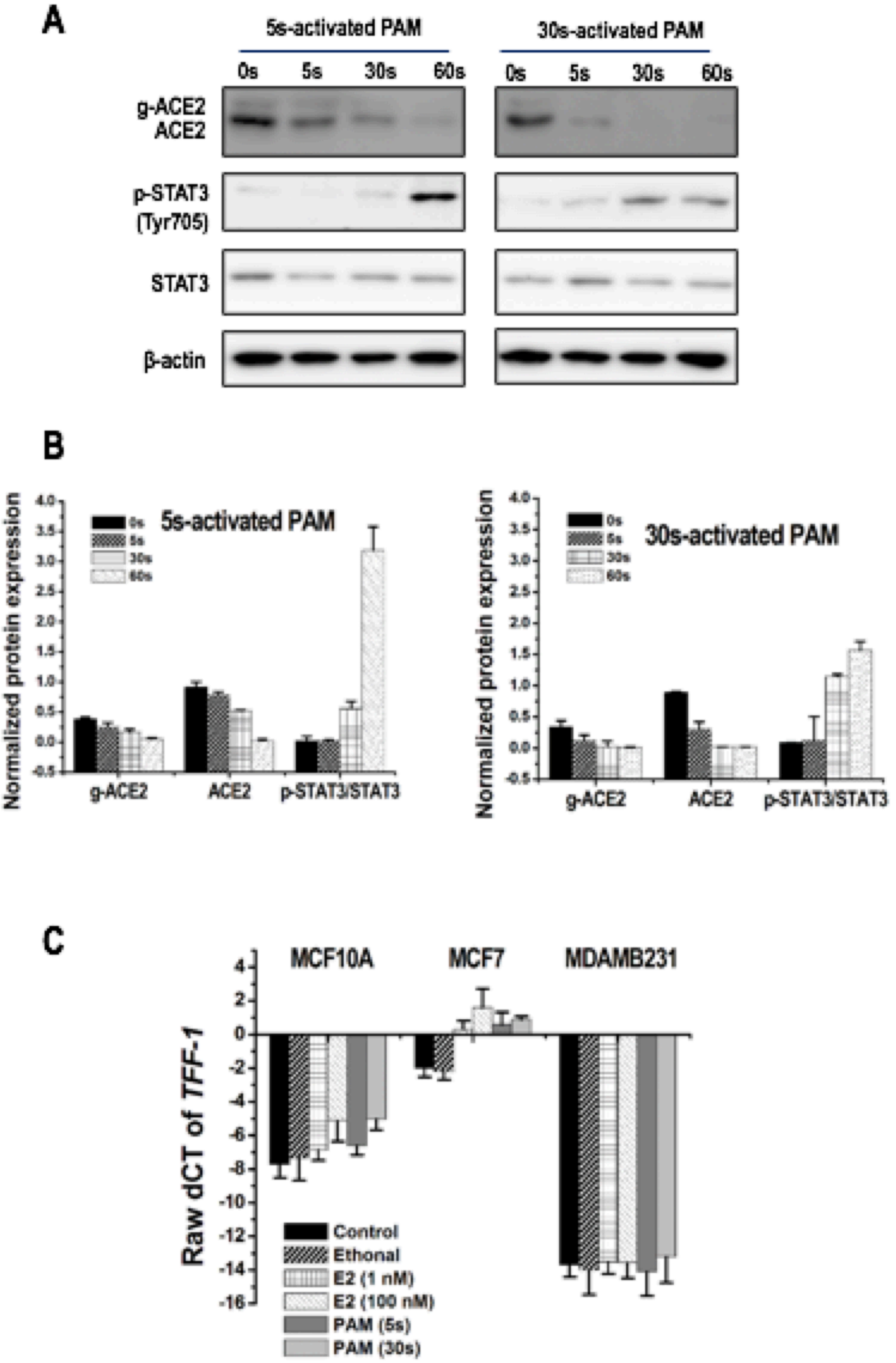

Figure S7. Additional images showing the dosing effect of PAM and estradiol on ACE2 and the ERα/phospho-STAT3(Tyr705) axis. (A) Western blot and (B) quantification of ACE2, STAT3, phospho-STAT3(Tyr705) expression by MCF7 cells incubated with 5 s- and 30 s- activated PAM for different durations. (C) RT-qPCR results showing expression of TFF-1 (a highly sensitive ER-response reporter) after treatment with different doses of PAM and estradiol in MCF10A cells, MDA-MB-231 cells and MCF-7 cells. In (C), TFF-1 raw Ct (cycle threshold) values are defined as the number of cycles required for the fluorescent signal to cross the threshold (*i.e.* exceed background level). Ct levels are inversely proportional to the amount of target nucleic acid in the sample (*i.e.*, the lower the Ct level the greater the amount of target nucleic acid in the sample); a dCt >1 is indicative of abundant target mRNA in the sample; -10<dCt <1 is indicative of moderate amounts of target mRNA; dCt<-10 is indicative of minimal amounts of target mRNA that could be due to environmental contaminations. Ethanol, the solution used for diluting E2, was used as a control in (C). 30 min was used for both E2 and PAM treatment in the RT-PCR testing TFF-1 gene expression.

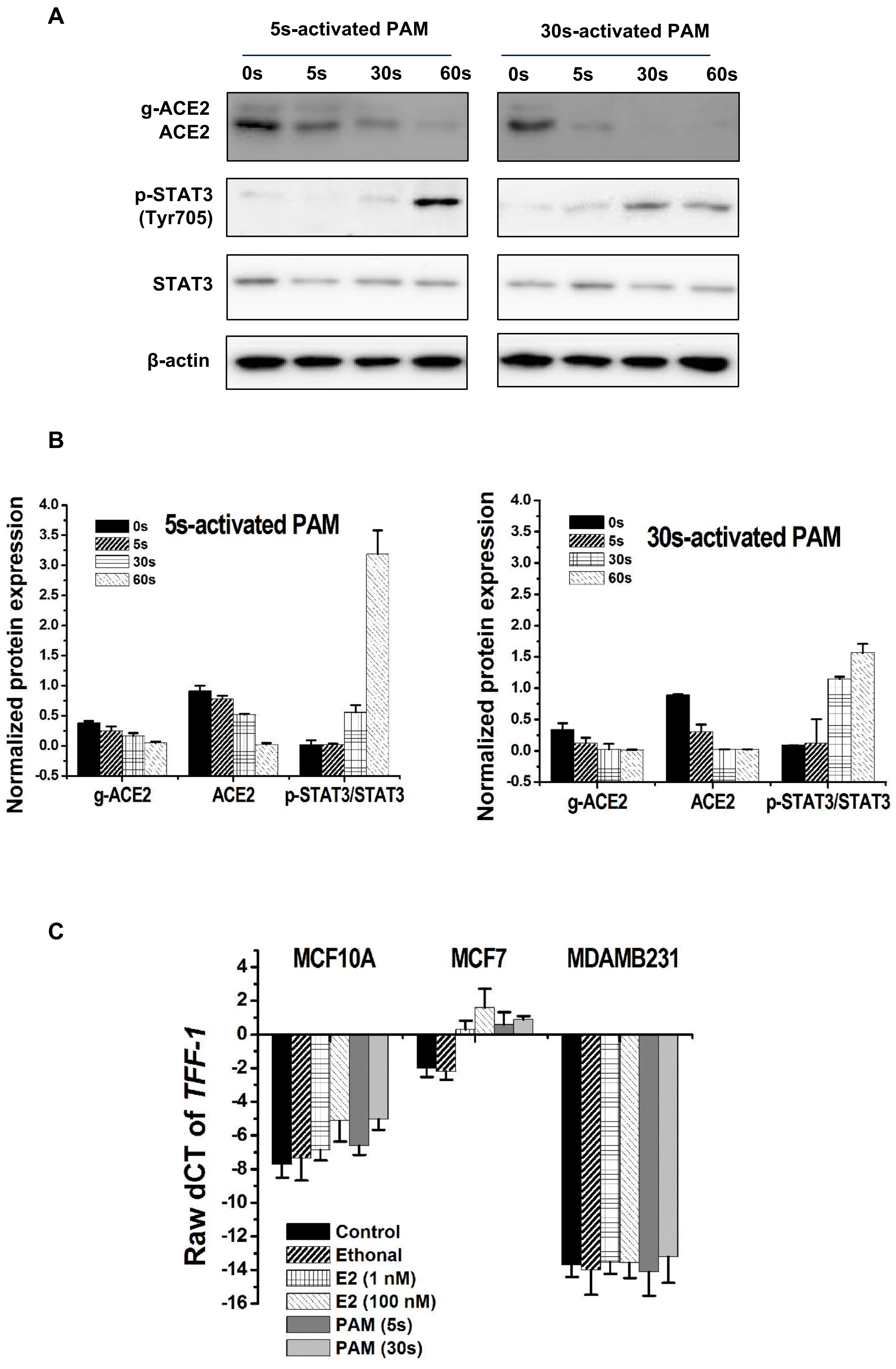

A
5s-activated PAM
30s-activated PAM
0s
5s
30s
60s
g-ACE2
ACE2
p-STAT3
(Tyr705)
STAT3
β-actin
B
Normalized protein expression
5s-activated PAM
30s-activated PAM
0s
5s
30s
60s
4.0
3.5
3.0
2.5
2.0
1.5
1.0
0.5
0.0
-0.5
g-ACE2
ACE2
p-STAT3/STAT3
C
MCF10A
MCF7
MDAMB231
Raw dCT of TFF-1
4
2
0
-2
-4
-6
-8
-10
-12
-14
-16
Control
Ethonal
E2 (1 nM)
E2 (100 nM)
PAM (5s)
PAM (30s)

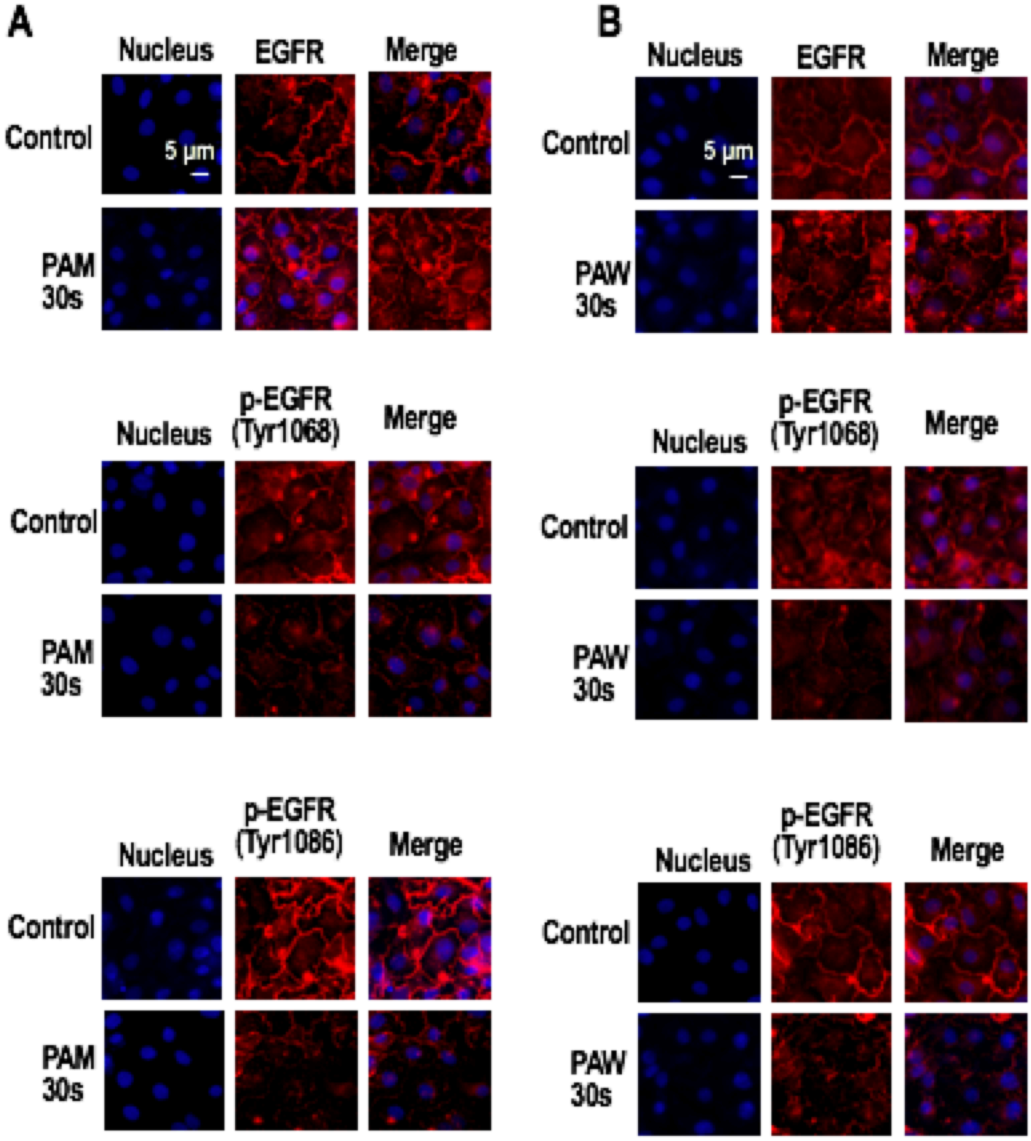

Figure S8. Additional immunofluorescence images showing EGFR(Tyr1068) and EGFR(Tyr1086) expression and cell localization after indirect CAP exposure for 30s. (A) Immunofluorescence image showing EGFR(Tyr1068) and EGFR(Tyr1086) location after indirect (A) PAM and(B), PAW treatment for 30 s. Scale bar=8 µm.

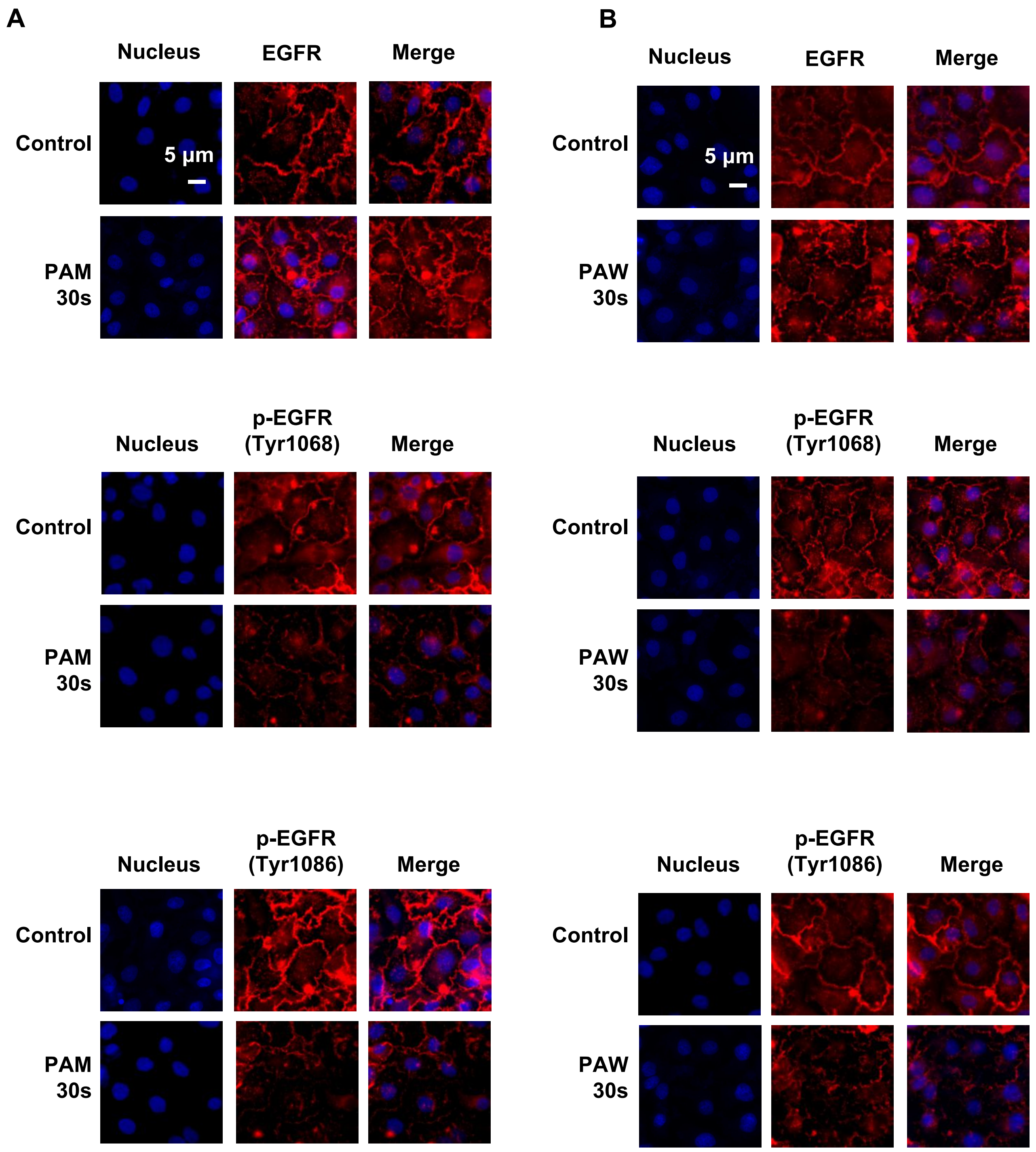
A
Nucleus
EGFR
Merge
Control
5 μm
PAM
30s
Nucleus
p-EGFR
(Tyr1068)
Merge
Control
PAM
30s
Nucleus
p-EGFR
(Tyr1086)
Merge
Control
PAM
30s
B
Nucleus
EGFR
Merge
Control
5 μm
PAW
30s
Nucleus
p-EGFR
(Tyr1068)
Merge
Control
PAW
30s
Nucleus
p-EGFR
(Tyr1086)
Merge
Control
PAW
30s
